# Identification and characterization of the TmSnRK2 family proteins related to chicoric acid biosynthesis in *Taraxacum mongolicum*

**DOI:** 10.1186/s12864-025-11460-w

**Published:** 2025-03-20

**Authors:** Qun Liu, Zhiqing Wu, Changyang Yu, Xiwu Qi, Hailing Fang, Xu Yu, Li Li, Yang Bai, Dongmei Liu, Zequn Chen, Guoyin Kai, Chengyuan Liang

**Affiliations:** 1 Institute of Botany, Jiangsu Province and Chinese Academy of Sciences (Nanjing Botanical Garden Mem. Sun Yat-Sen), Nanjing, 210014 China; 2 Jinhua Academy, Zhejiang International Science and Technology Cooperation Base for Active Ingredients of Medicinal and Edible Plants and Health, College of Pharmacy, Zhejiang Chinese Medical University, Hangzhou, 310053 Zhejiang China; 3 Jiangsu Key Laboratory for the Research and Utilization of Plant Resources, Nanjing, 210014 China

**Keywords:** SnRK2s, *T. mongolicum*, LC-MS, Transcriptomic data, Chicoric acid

## Abstract

**Background:**

*Taraxacum mongolicum* is rich in phenolic acids and is widely utilized in food and medicine globally. Our previous research demonstrated that the abscisic acid (ABA) hormone significantly enhances chicoric acid accumulation in *T. mongolicum*. SNF1-related protein kinase 2s (SnRK2s) are extensively involved in ABA signaling and have the potential to regulate the biosynthesis of phenolic acids.

**Results:**

In this study, liquid chromatography-mass spectrometry (LC-MS) and transcriptomic analyses revealed that the TmbZIP1-Tm4CL1 pathway plays a crucial role in the transcriptional regulation of chicoric acid biosynthesis. Seven TmSnRK2s were identified in *T. mongolicum* and classified into three groups. Analysis of the TmSnRK2s promoters (2000 bp in length) indicated that the three most prevalent stress-related elements were ABA, methyl jasmonate (MeJA), and light. ABA treatments (0 h, 2 h, 4 h, 8 h, and 24 h) showed that all seven TmSnRK2s were significantly modulated by ABA, with the exception of SnRK2.7. TmSnRK2.2, TmSnRK2.3, TmSnRK2.6, and TmSnRK2.7 were localized in both the cytoplasm and nucleus, whereas TmSnRK2.1 and TmSnRK2.5 were exclusively observed in the cytoplasm. Yeast two-hybrid (Y2H) and bimolecular fluorescence complementation (BiFC) assays indicated that TmSnRK2.1, TmSnRK2.3, TmSnRK2.6, and TmSnRK2.7 interact with TmbZIP1. The motifs ‘Q(S/G)(V/D)(D/E)(I/L)××I(I/V)×EA’ and ‘D×(D/ED××D)’ are identified as the core sites that facilitate the binding of TmSnRK2s to TmbZIP1. Dual-luciferase reporter assays demonstrated that TmSnRK2.3 and TmSnRK2.6 enhance the stability of TmbZIP1 binding to proTm4CL1.

**Conclusion:**

These findings enhance our understanding of the specific roles of certain members of the TmSnRK2 family in the biosynthesis pathway of chicoric acid.

**Supplementary Information:**

The online version contains supplementary material available at 10.1186/s12864-025-11460-w.

## Background

Dandelion, also known as *Taraxacum* spp, is a well-known Asteraceae plant that is distributed worldwide, with more than 2000 species [1]. The *Taraxacum* name derives from the Greek terms “taraxis” and “akeomai,” and 10th/11th centuries Arab physicians mentioned the evidence of its therapeutic effect on liver and spleen diseases, whereas dandelion roots and leaves were widely used in Europe to treat gastrointestinal disorders [2]. The European Scientific Cooperation for Plant Therapeutics recommends that dandelion roots can be used to restore liver and gallbladder function and reduce indigestion. According to the U.S. Food and Drug Administration, dandelions are “generally recognized as safe” for food without causing allergic reactions, diarrhea, or gastrointestinal upset. Breastfed infants can use it without harm during lactation [3, 4]. Phytochemicals in dandelions include phenolic acids, polysaccharides, terpenes, etc; taraxasterol has anti-tumor effect, polysaccharides have antioxidant and immunomodulatory effects, and chlorogenic acid and chicoric acid have anti-oxidative and anti-viral effects [5–7]. The application of patented Chinese medicines in traditional Chinese medicine treatment has continuously increased in recent decades. Among them, Pudilan Xiaoyan Oral Liquid (PDL), with dandelion as a raw material, is a typical representative of Chinese patented medicine that helps fight viral infections even COVID-19 [8–10]. In addition to being used in medicine, dandelion leaves, roots, and flowers are processed into different foods. Many dandelion products have appeared in the market, including dandelion root/leaf/flower tea, effervescent tablets, and other products obtained through different processing techniques [11–13]. However, the biosynthetic mechanism of the main secondary metabolites, such as chicoric acid (an index medicinal ingredient) in dandelions, is still unclear [14], and relevant reports on the precursor compounds for chicoric acid biosynthesis in different tissue parts are still lacking [11, 15, 16].

The accumulation of plant secondary metabolites in various tissues exhibits considerable variation. Differential transcriptomic analyses of tissues can be employed to identify and compare genes that are differentially expressed across different tissues [17, 18]. This includes genes involved in the biosynthesis of compounds such as tanshinone, notoginseng triterpenes, ginsenosides, and atractylodes sesquiterpenes, which are specifically accumulated in roots; artemisinin and menthol, which are specifically accumulated in glandular hairs; and anthocyanins and luteolin, which are specifically accumulated in flowers [17–19]. Previous studies have indicated that the hormone ABA can enhance chicoric acid accumulation in dandelions by promoting the expression of chicoric acid biosynthetic enzyme genes in *T. mongolicum*. Furthermore, the TmbZIP1-Tm4CL1 pathway plays a crucial role in ABA-mediated regulation of chicoric acid biosynthesis in dandelions [20]. Current research has produced extensive transcriptome data derived from the application of MeJA and ABA hormones across various species, including *Taraxacum* and *Artemisia annua*. These studies primarily focus on the effects of exogenous hormone treatment on plant responses. However, challenges remain regarding the selection of hormone concentrations and the establishment of feedback regulation mechanisms, which cannot be effectively circumvented [21, 22]. The tissue differential transcriptomic analyses can reveal gene expression differences caused by tissue differentiation [23, 24]. Furthermore, highly differentiated tissues show differences in the types and expression levels of genes, making it easier to search for major regulatory effectors and signaling pathways [24, 25]. Therefore, combined analysis of the tissue differential transcriptome and LC-MS is beneficial for obtaining more systematic and complete omics data to study the relationship between secondary metabolite accumulation and gene expression in *T. mongolicum*.

The sucrose non-fermenting 1-related protein kinase (SnRK) family belongs to Ser/Thr protein kinases that are widely present in plants that are involved in the transduction of various signaling pathways (ethylene, gibberellin (GA), salicylic acid (SA), MeJA signals, etc.) in plants, and play essential roles in plant stress resistance, including drought stress and salt stress [26, 27]. Based on their domains, SnRKs consists of three subfamilies, including SnRK1, SnRK2 and SnRK3. Among them, SnRK2s are only distributed in plants, have a number of functions, and are known to be important regulators of ABA signaling. ABA hormones can inhibit the formation of the PP2C-SnRK2s protein complex and induce the release SnRK2s proteins, which phosphorylate transcription factors (TFs) to regulate downstream pathways [28]. In *A. annua*, AaSnRK2.6 which binds to the AabZIP1 transcription factor, positively regulates artemisinin biosynthesis [29, 30]. In *Salvia miltiorrhiza*, SmSnRK2.6 can regulate the expression of *SmPAL*, *Sm4CL*, *SmHPPR*, and other genes to increase the biosynthesis of phenolic compounds; SmSnRK2.3/2.6 can interact with SmAREB1 [31]; and SnRK2.3/2.6/2.10 can bind to SmbZIP2 and negatively regulate salvianolic acid biosynthesis [32]. Previous research has shown that a member of SnRK2s from *T. mongolicum* (TmSnRK2s) named TmSnRK2.6 can interact with TmbZIP1 in yeast and tobacco which is proved that TmSnRK2s are important in regulating secondary metabolic biosynthesis [20]. However, the basic information (gene structure, promoter structure et al.) and functions of SnRK2s family members in *T. mongolicum* have not been reported. Whether TmSnRK2s affect the binding of TmbZIP1 to the downstream Tm4CL1 promoter to regulate chicoric acid biosynthesis in *T. mongolicum* requires further investigation.

In this study, we found that chicoric acid and its related compounds were higher in the leaves than in the flowers and roots. The transcriptomes of *T. mongolicum* in different tissues were sequenced, generated a total of 65.09 Gb clean data and 77,035 unigenes. TmbZIP1-Tm4CL1 is still an important pathway in the transcriptional regulation of chicoric acid biosynthesis. Seven SnRK2s were cloned and analyzed using the *T. mongolicum* genome, ABA hormone-induced temporal- and tissue-specific transcriptome from this study. Phylogenetic relationships, gene structures, and conserved motif composition of TmSnPK2s were determined; cis-elements in the promoters of TmSnRK2s were analyzed; the expression levels of *TmSnRK2s* in different tissues and under ABA-hormones treatment were detected through transcriptomic analysis and qRT-PCR; and the subcellular localization of TmSnRK2 proteins was determined. Y2H and BiFC assays indicated that TmSnRK2.1/2.3/2.6/2.7 can interact with TmbZIP1. The ‘Q(S/G)(V/D)(D/E)(I/L)××I(I/V)×EA’ and ‘D×(D/ED××D)’ motifs are the core sites that determines the binding of TmSnRK2s to TmbZIP1, whereas the ‘protein kinase activating’ domain of *TmSnRK2.3* can interact with TmbZIP1. Dual-luciferase reporter assay experiments revealed that TmSnRK2.3/2.6 can promote the stability of TmbZIP1 binding to proTm4CL1 in tobacco. Our study extends our understanding of the TmSnRK2 family in *T. mongolicum* and provides useful information for further evaluation of TmSnRK2 functions.

## Results

### Chicoric acid and its precursor compounds determination in different tissues in T. mongolicum

Chicoric acid is a chlorogenic acid downstream products that is well-known for its bioactivity in *T. mongolicum*. 11 chicoric acid-related compounds (5-O-caffeoylshikimic acid, caffeic acid, caftaric acid, chlorogenic acid, D-chicoric acid, isochlorogenic acid A, L-chicoric acid, L-phenylalanine, *p*-coumalic acid, quinic acid, and tartaric acid) were identified through LC-MS metabolite analysis (Table 1, Fig. S1). As shown in Tables 1 and 5-O-caffeoylshikimic acid, chlorogenic acid, and isochlorogenic acid A were the highest in the flowers, quinic acid was the highest in the roots, and tartaric acid, L-phenylalanine, caftaric acid, *p*-coumalic acid, caffeic acid, L-chicoric acid, and D-chicoric acid were the highest in the leaves. However, 5-O-caffeoylshikimic acid, p-coumalic acid, and caffeic acid were not detected in the roots, whereas l-chicoric acid was not detected in the flowers. In addition, 5-O-caffeoylshikimic acid was the highest in the leaves and *p*-coumaric acid was the highest in the flowers, which was not significantly different from that in other tissues. In general, the leaves of *T. mongolicum* accumulate high contents of chicoric acid and its related compounds. Subsequently, we considered useful to investigate the biosynthetic mechanisms of chicoric acid regulatory pathways using transcriptome sequencing.

**Table 1:** Summary of phenolic acid compounds identified by LC-MS from three different tissues in *T. mongolicum*

Name	tR (time)	Formula	Detected mass (m/z)	Root	Leaf	Flower
5-O-caffeoylshikimic acid	1.08	C_16_H_16_O_8_	335	NA	4,293,770	**5**,**949**,**565**
tartaric acid	1.14	C_4_H_6_O_6_	149	148,741,592	**461**,**956**,**487**	255,595,891
quinic acid	1.67	C_7_H_12_O_6_	191	**1**,**790**,**312**,**350**	650,049,582	58,104,114
L-phenylalanine	2.20	C_9_H_11_NO_2_	164	36,914,279	**73**,**808**,**346**	26,399,698
caftaric acid	12.34	C_13_H_12_O_9_	311	49,185,566	**414**,**459**,**731**	72,392,367
*p*-coumalic acid	15.64	C_6_H_4_O_4_	139	NA	**15**,**626**,**781**	13,597,985
caffeic acid	17.66	C_9_H_8_O_4_	179	NA	**40**,**390**,**192**	14,809,587
chlorogenic acid	19.4	C_16_H_18_O_9_	353	188,571,346	510,649,344	**762**,**675**,**537**
L-chicoric acid	24.41	C_22_H_18_O_12_	473	9,875,936	**1**,**706**,**070**,**695**	NA
D-chicoric acid	29.86	C_22_H_18_O_12_	473	537,100,170	**4**,**057**,**936**,**374**	978,947,266
isochlorogenic acid A	32.61	C_25_H_24_O_12_	515	14,434,3609	152,238,351	**258**,**167**,**407**

Note: Signal-to-noise ratios (S/*N* > 3) were used in qualitative experiments for compounds to determine the presence or absence of compounds

### Transcriptome sequencing and de Novo assembly of T. mongolicum

**Fig. 1:**
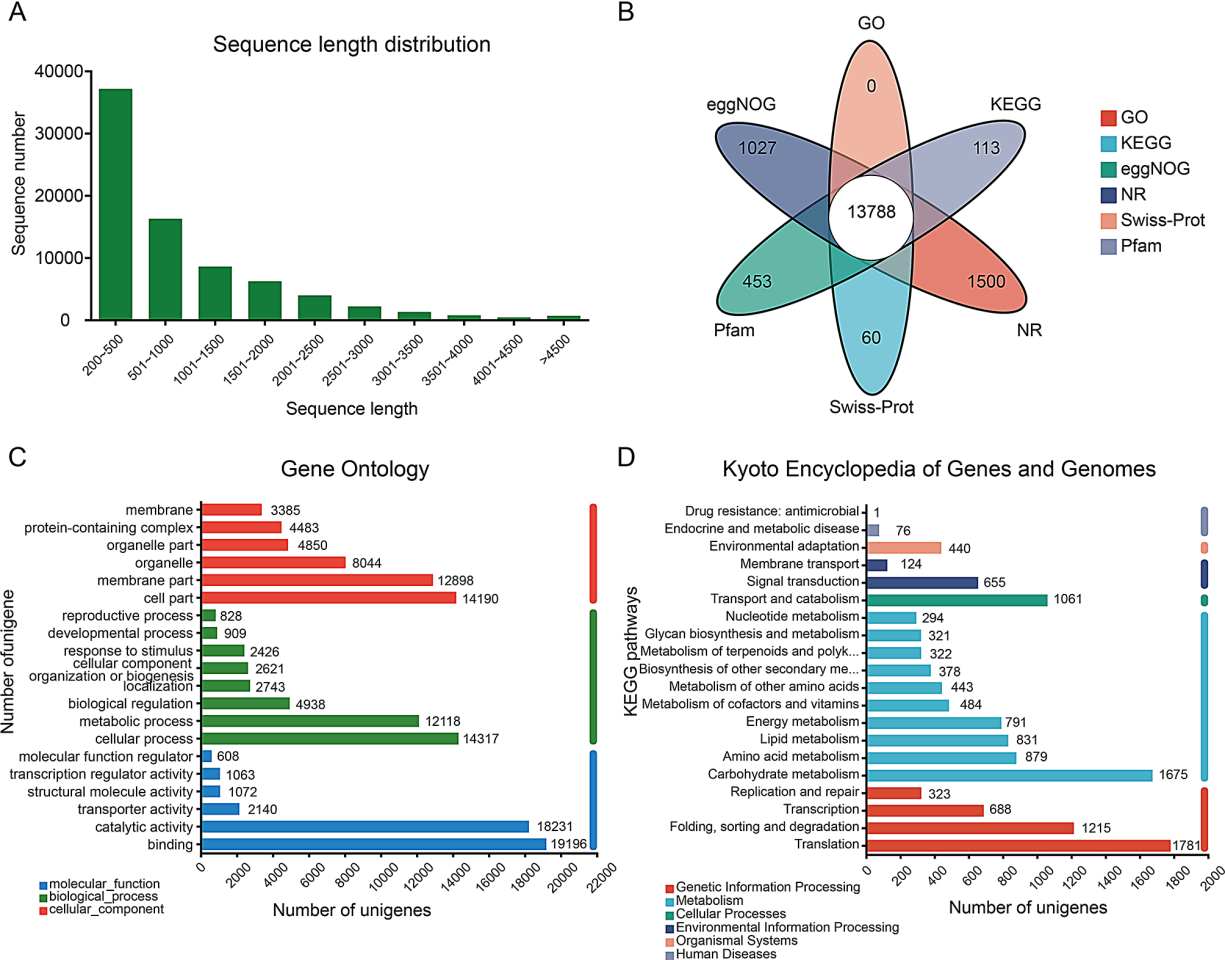
Transcriptome assembly and annotation. **A**. Length distribution of the unigenes. **B**. Venn diagram illustrating the functional annotation of six public protein databases: eggNOG, Gene Ontology (GO), Kyoto Encyclopedia of Genes and Genomes (KEGG), Non-Redundant (NR), Swiss-Prot, and Pfam. **C**. Distribution of GO annotations for all unigenes, classified into three primary categories: biological process, cellular component, and molecular function. **D**. KEGG annotation and classification of unigenes

To investigate the biosynthetic mechanism of chicoric acid, three different tissues (leaf, root, and flower; three biological replicates) of *T. mongolicum* were selected for transcriptome sequencing. Therefore, nine cDNA libraries were constructed and sequenced using Illumina paired-end sequencing technology. 65.09 Gb clean data were acquired from leaf, root, and flower tissues (49,692,629, 49,185,947 and 48,321,138 average clean reads, respectively). Table S2 shows an overview of the raw and clean data obtained using *T. mongolicum* transcriptome sequencing. A total of 128,250 unigenes were identified, collected and organized using the Trinity program. The length distribution of unigenes results indicated that 31% unigenes lengths were > 1 kb, whereas the unigene GC content was within 44–46% (Fig. 1A, Table S3). The longest unigene sequence was 20,797 bp, whereas the shortest was 201 bp, followed by an average length of 918.96 bp. The raw data were submitted to the Short Read Archive (SRA; accession number PRJNA861012) and found in the NCBI database.

### Functional classification and annotation of unigenes

As shown in Table S4, 33,635 (43.66%) unigenes were matched in the GO database, whereas 21,091 (27.38%) matched in KEGG, 40,360 (52.39%) matched in COG, 40,732 (52.87%) matched in NR, 33,933 (40.05%) matched in Swiss-Prot, and 34,035 (44.18%) matched in Pfam databases. In addition, 13,788 unigenes were annotated in 6 databases (NR, KEGG, COG, Prot, Swiss Prot, Pfam), there are 1,027 unigenes annotated in COG and 113 in KEGG, 1500 in NR, 60 in Swiss Prot, 453 in Pfam (Fig. 1B). The NR database had the largest number of annotations among the six databases, and *Lactuca sativa* (25,580), *Rhodamnia argentea* (4,800), and *Cynara cardunculus* (2,090) had the three closest matches. In the NR database, the e-value distribution suggested that 70.09% of the annotated unigenes reached e-value < 10^− 30^ (Fig. S2).

The KEGG, GO, and COG databases were employed to categorize the predicted unigenes in *T. mongolicum*. The GO terms encompassed 33,635 unigenes based on their functions, which were organized into three primary categories: biological processes, molecular functions, and cellular components (Fig. 1C). In the molecular function category, the three most frequent terms were “binding” (19,196), “catalytic activity” (18,231), and “transporter activity” (2,140). Within the biological process category, the predominant terms included “cellular process” (14,317), “metabolic process” (12,118), and “biological regulation” (4,938). For the cellular component category, the primary terms were “cell part” (14,190), “membrane part” (12,898), and “organelle” (8,044). Subsequently, a total of 21,091 unigenes (27.38%) were assigned to 20 KEGG pathways, primarily categorized into “metabolism” (4,743), “genetic information processing” (4,467), “environmental information processing” (1,870), “cell process” (1,061), and “organismal systems” (440). In the KEGG database, the three most representative pathways were “translation” (1,781), “carbohydrate metabolism” (1,675), and “folding, sorting and degradation” (1,215) (Fig. 1D). Additionally, a total of 40,360 unigenes (52.39%) were classified into 23 COG categories, with the five most common groups being “posttranslational modification, protein turnover, chaperones” (2,630), “signal transduction mechanisms” (2,463), “transcription” (2,225), “intracellular trafficking, secretion, and vesicular transport” (1,804), and “replication, recombination and repair” (1,723) (Fig. S3).

### Differential expression analysis of unigenes in T. mongolicum

**Fig. 2:**
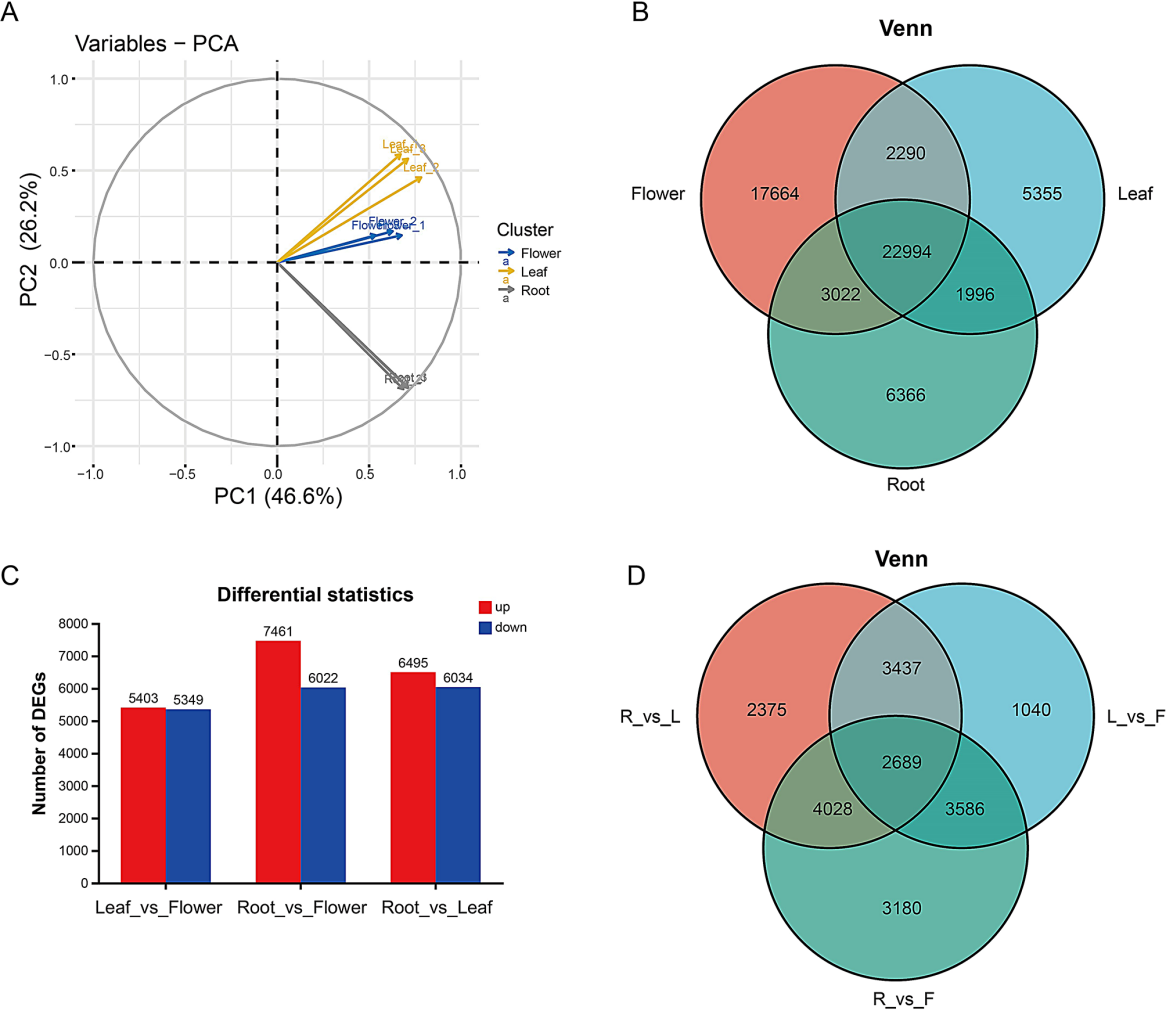
Differentially expressed genes analysis of tissue samples (leaf, root, and flower) in *T. mongolicum*

To identify differentially expressed genes (DEGs), Fragments Per Kilobase per Million (FPKM) values and the distribution of assembled unigenes were calculated in *T. mongolicum*. Scree Plot was used for principal component analysis (PCA), among which the first principal component (PC1) explains approximately 46.6% of the total variance, while PC2 sharing 26.2% (Fig S4A-D). PC1 and PC2 including 72.8% total variance which indicated that the leaf, flower and root samples have good repeatability and significant differences (Fig. 2A). A total of 22,994 unigenes were expressed in all three tissues; 5,355 unigenes were specifically expressed in the leaves, 17,664 unigenes were specifically expressed in the flowers, and 6,366 unigenes were specifically expressed in the roots (Fig. 2B).

A, Principal component analysis (PCA) analysis of the 9 samples; B, The venn diagram shows the number of expressed genes (FPKM > 1) in different samples; C, Differential statistics of DEGs in comparison of root, flower, and leaf samples; D, The venn diagram shows the numbers of DEGs in comparison of Root_vs_Leaf, Root_vs _Flower, and Leaf _vs _Flower groups.

Due to the high concentration of chicoric acid and its related compounds in the leaves of *T. mongolicum*, differential expression analyses were performed comparing leaves to roots and leaves to flowers to investigate the biosynthetic mechanisms of chicoric acid. Using the criteria of *p*-adjusted < 0.05 and log2FC ≥ 1, we compared unigene expression levels in the Root_vs_Leaf, Root_vs_Flower, and Leaf_vs_Flower groups to analyze DEGs. A total of 30,302 DEGs were identified for statistical analysis, revealing that the Root_vs_Flower comparison yielded the highest number of DEGs, with 7,461 DEGs upregulated in the roots and 6,022 DEGs upregulated in the flowers (Fig. 2C). Among these, unigenes involved in photosynthesis, plant hormone signal transduction, and phenylpropanoid biosynthesis exhibited higher expression levels in the leaves (Fig. S4). A total of 2,689 DEGs were significantly differentially expressed across the Root_vs_Leaf, Root_vs_Flower, and Leaf_vs_Flower comparisons, while 2,375, 3,180, and 1,040 DEGs were significantly differentially expressed only in the Root_vs_Leaf, Root_vs_Flower, and Leaf_vs_Flower groups, respectively (Fig. 2D and Fig. S5). Regarding chicoric acid biosynthesis, the intersection of DEGs from the Root_vs_Leaf and Leaf_vs_Flower comparisons identified 6,126 DEGs that warrant further investigation.

### Analysis of Chicoric acid biosynthesis genes and related TFs

**Fig. 3:**
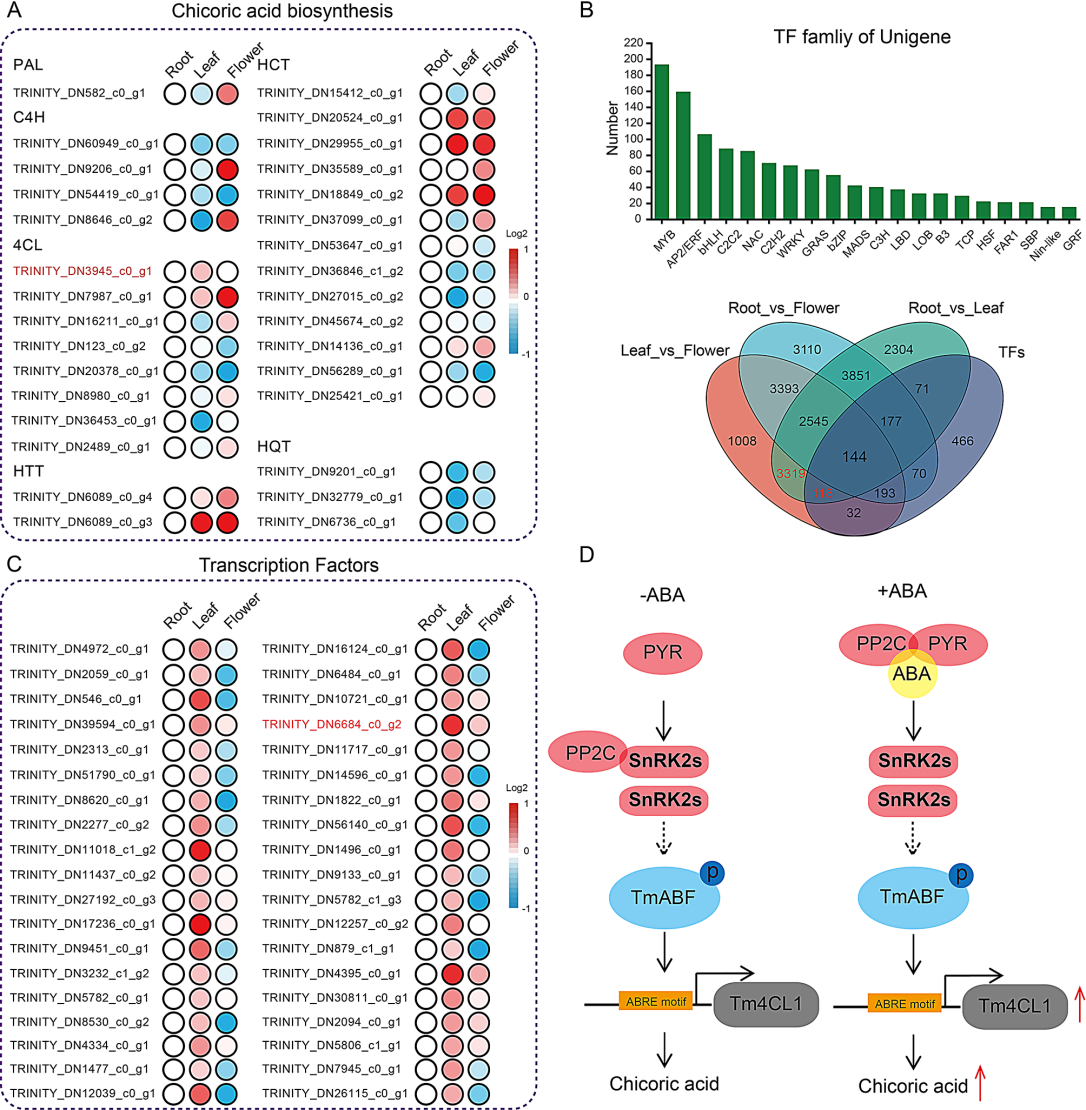
Analysis of chicoric acid biosynthesis genes and TFs in *T. mongolicum*

Recently, 41 chicoric acid biosynthesis genes were reported in the ABA-induced transcriptome data [20]. We found 31 chicoric acid biosynthesis DEGs in different tissues. Among them, Tm4CL1 (DN3945_c0_g1; Leaf > root/flower) was the most related and examined using qRT-PCR (Fig. 3A). Only one PAL member was present in all three tissues and was differentially expressed, whereas four C4H members were found more frequently than previously reported [20].

We further analyzed the TFs in different tissues and found 20 families containing 1271 members, with the three largest TF families being MYB, AP2/ERF, and bHLH (Fig. 3B). We added the 1271 gene sets of TFs into the DEGs of root vs. leaf, root vs. flower, and leaf vs. flower groups to create a new gene set. Tm4CL1 belongs to the (3319) gene set; therefore, we further analyzed this gene set (118). As shown in Fig. 3C, after removing those with FPKM < 1, 38 TFs were closely related to Tm4CL1, among which DN6654_c0_g2 (TmbZIP1, also named ABRE-binding factor, short for ABF) was studied [20]. The rest unevaluated 37 TFs potentially regulate chicoric acid biosynthesis in *T. mongolicum*. As reported for *Arabidopsis*, AtSnRK2s often phosphorylate ABFs to activate downstream pathways (Fig. 3D). However, research on the SnRK2s family members in *T. mongolicum* is in its early stages.

A, Analysis of chicoric acid biosynthesis genes in different tissues of *T. mongolicum*. B, Analysis of transcription factors in different tissues of *T. mongolicum*. 3319 red marked unigenes (maining functional genes) reflects the level of expression were leaf > root/flower; 118 red marked TFs members reflects the level of expression were leaf > root/flower. C, Heat map analysis of 38 TFs related to chicoric acid biosynthesis in different tissues of *T. mongolicum*. D, Possible pathway analysis of TmbZIP1 (TmABI5) involvement in chicoric acid biosynthesis.

### Analysis of TmSnRK2s in T. mongolicum

**Fig. 4:**
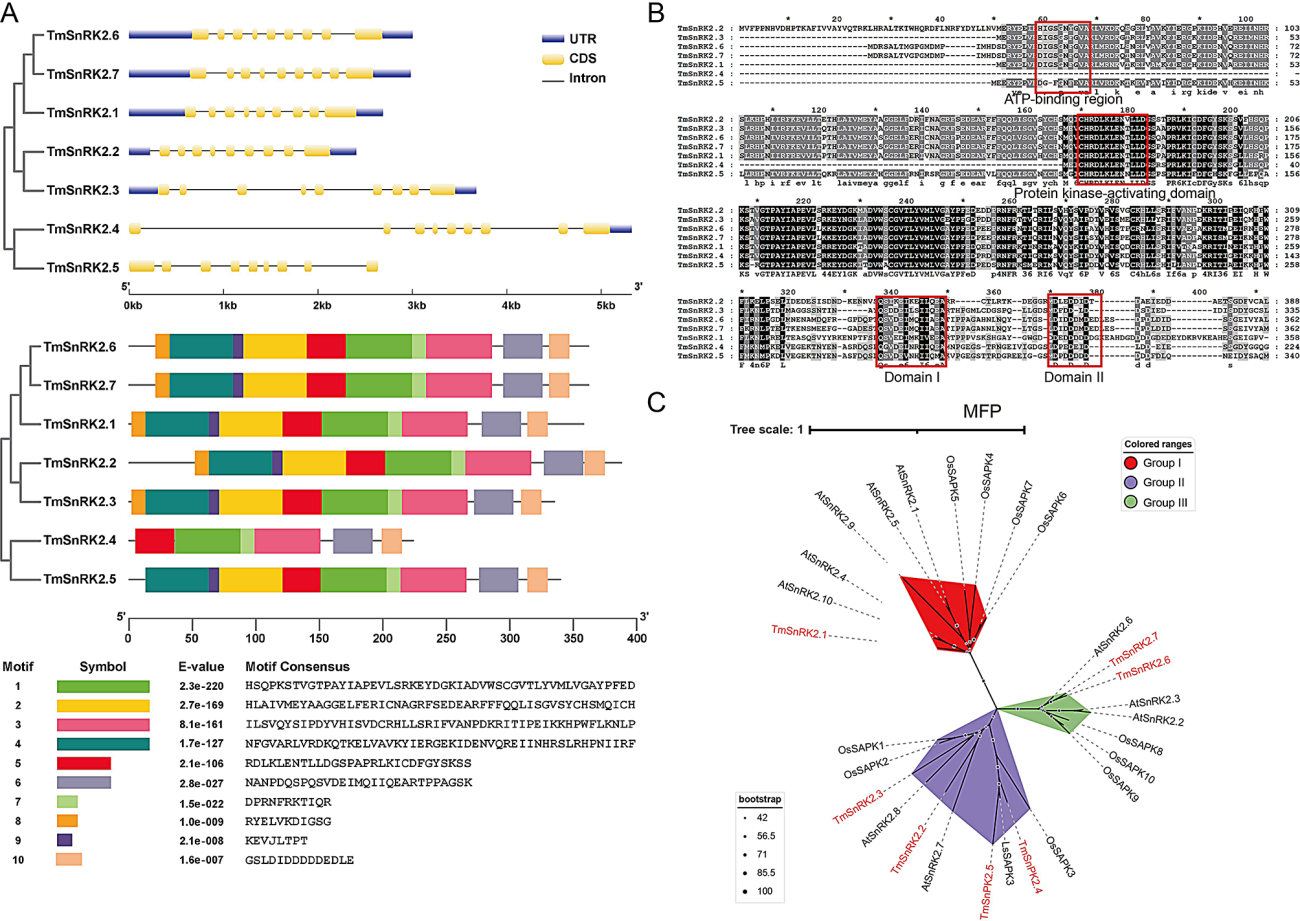
Bioinformatics analysis of TmSnRK2s

Seven SnRK2 members were identified in the genome of *T. mongolicum*. The ABA-induced and tissue transcriptomes were previously reported (Table S5). In this study, we analyzed the phylogenetic relationships, genetic structures, and conserved motif compositions of TmSnRK2s. The exon-intron organization of TmSnRK2s revealed that all members contain nine introns, with lengths ranging from 2400 to 5300 bp (Fig. 4A). The conserved motif analysis indicated that TmSnRK2.1, TmSnRK2.2, TmSnRK2.3, TmSnRK2.6, and TmSnRK2.7 possess ten motifs, TmSnRK2.5 has nine motifs, and TmSnRK2.4 has only six motifs. Analysis of the promoter cis-elements of TmSnRK2s revealed the presence of 12 cis-elements, among which the ABRE, CGTCA/TGACG, TCA, and GT1 motifs were the most abundant (Table 2). These cis-elements are strongly associated with ABA, MeJA, and SA hormone signaling, as well as light signal transduction, indicating that TmSnRK2s are extensively involved in various signaling pathways, consistent with previous findings in other plants [33]. Based on these results, we performed an amino acid alignment of TmSnRK2 members and found that the ATP-binding region, protein kinase-activating domain, Domain I, and Domain II are present in TmSnRK2.1, TmSnRK2.2, TmSnRK2.3, TmSnRK2.5, TmSnRK2.6, and TmSnRK2.7, while TmSnRK2.4 lacks the ATP-binding region (Fig. 4B). The phylogenetic comparison of the TmSnRK2 family from *T. mongolicum* with those from *A. thaliana* and *O. sativa L*. indicates that TmSnRK2s can be categorized into three groups: TmSnRK2.1 belongs to Group I, TmSnRK2.2, TmSnRK2.3, TmSnRK2.4, and TmSnRK2.5 belong to Group II, and TmSnRK2.6 and TmSnRK2.7 belong to Group III (Fig. 4C).

**Table 2:** cis-elements in promoters of SnRK2s

Cis element	Functional annotation	TmSnRK2s						
		TmSnRK2.1	TmSnRK2.2	TmSnRK2.3	TmSnRK2.4	TmSnRK2.5	TmSnRK2.6	TmSnRK2.7
ABRE	Abscisic acid responsiveness	1	3		1	3	1	1
CGTCA/TGACG	MeJA-responsiveness	1	1		1	1	1	3
ERE	ethylene-responsive element		2	1		2	1	1
GARE	gibberellin-responsive						1	1
TGA element	auxin-responsive element				1			
TCA element	salicylic acid responsiveness	7	1	1	1	3	1	
LTR	low-temperature responsiveness	1			1	2		
MBS	MYB binding site involved in drought-inducibility	3					1	
P-BOX	gibberellin-responsive element	1			1	1	1	
G-box	light-responsiveness		4		2	4		1
GT1 motif	light responsive element		1	1	5	3	1	1
MRE	MYB binding site involved in light responsive		2		1			

(A) Phylogenetic relationships, exon-intron organization, and motif analysis of TmSnRK2s are presented. Exons and introns are represented by filled boxes and single lines, respectively. (B) Amino acid alignment of TmSnRK2 members in *T. mongolicum*, with domains underlined in black. The TmSnRK2s highlighted in red indicate the members identified in this study. (C) Construction of the phylogenetic tree for SnRK2 family members from *T. mongolicum* compared to those from *A. thaliana* and *O. sativa* L.SnRK2 members (AtSnRK2.1-AtSnRK2.10 accession number: P43292, Q39192.1, Q39193.1, P43291.1, Q9FFP9.1, Q940H6.1, Q9SMQ4.1, Q9M9E9.1, O64812.1, Q9C958.1; OsSAPK1-10:Q75LR7.1, Q0D4J7.1, P0C5D6.1, Q5N942.2, Q7XKA8.1, Q6ZI44.1, Q7XQP4.2, Q7Y0B9.1, Q75V57.1, Q75H77.1).

### Analysis of TmSnRK2 expression levels and subcellular localization in T. mongolicum

**Fig. 5:**
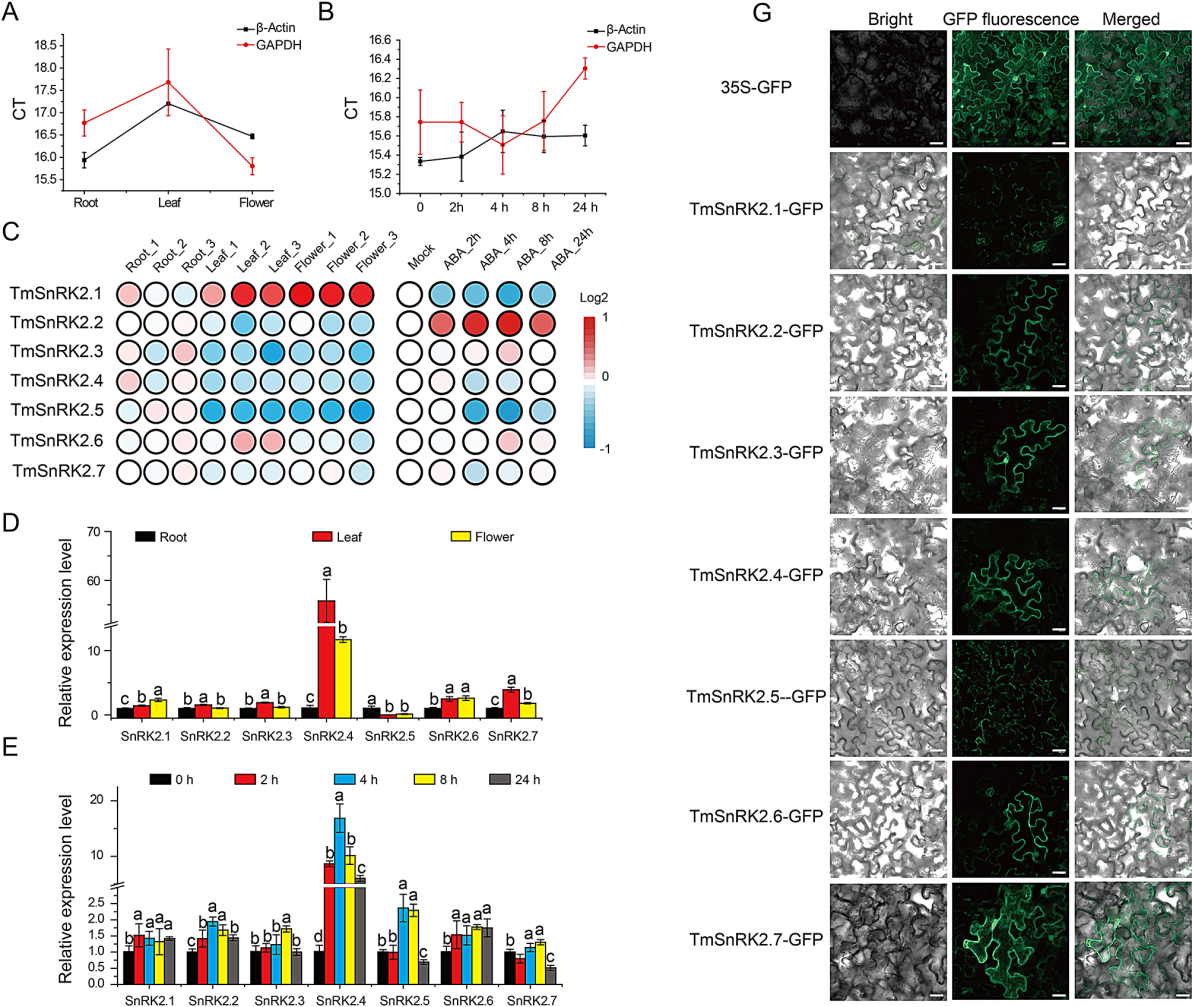
qRT-PCR Analysis and Subcellular Localization of TmSnRK2 Proteins

To further investigate the expression levels of TmSnRK2, we initially screened internal reference genes and compared the CT values of the β-actin and GADPH genes. The results indicated that in various tissues and ABA-treated *T. mongolicum* samples, the stability of β-actin and the CT value of GADPH ranged from 15 to 30. Notably, β-actin exhibited greater stability and was selected as the internal reference gene (Fig. 5A and B; Table S7).

A and B, The CT levels of β-Actin and GADPH in various tissues (root, leaf, and flower) and under ABA treatment; C, Heatmap of TmSnRK2s transcriptome analysis in differential tissues and under ABA hormone treatment in *T. mongolicum*; D, qRT-PCR analysis of TmSnRK2s in different tissues (root, leaf, and flower); E, qRT-PCR analysis of TmSnRK2s in ABA-treated leaves of *T. mongolicum* at different time points (0 h, 2 h, 4 h, 8 h, and 24 h); F, Subcellular localization of TmSnRK2 proteins in tobacco leaves. Different letters denote statistical significance (*p* < 0.05).

The heatmap of TmSnRK2 genes in three tissues of *T. mongolicum* (leaf, root, and flower) indicated that TmSnRK2.1 was highly upregulated in flowers, TmSnRK2.6 was upregulated in leaves, while TmSnRK2.5 was highly upregulated in roots (Fig. 5C). Notably, the qRT-PCR results revealed that TmSnRK2.2, TmSnRK2.3, TmSnRK2.4, and TmSnRK2.7 were highly expressed in the leaves which were different with the heatmap of TmSnRK2s transcriptome data (Fig. 5D). Furthermore, The expression pattern of TmSnRK2s in leaves induced by ABA hormones (0 h, 2 h, 4 h, 8 h, and 24 h) in our early transcriptome data indicates that TmSnRK2s significantly responds to ABA hormones (Fig. 5C; [20]). TmSnRK2.2, 2.3, 2.4, 2.5 and 2.7 exhibited an initial increase in expression followed by a decline, as observed in both transcriptome and qRT-PCR data (Fig. 5E). In contrast, TmSnRK2.1 displayed a trend of decreasing followed by increasing expression, while TmSnRK2.6 showed only increasing trends without significance. Additionally, the qRT-PCR results indicated that TmSnRK2.4 and TmSnRK2.5 were significantly induced by ABA treatment, which was inconsistent with the transcriptome data (Fig. 5C and E). Therefore, transcriptome data could be regarded as a preliminary reference and must be validated through qRT-PCR. The TmSnRK2-GFP vectors were individually transformed into *Agrobacterium* GV3101 for subcellular localization experiments, revealing that TmSnRK2.2, TmSnRK2.3, TmSnRK2.4, TmSnRK2.6, and TmSnRK2.7 localized to both the cytoplasm and nucleus, whereas TmSnRK2.1 and TmSnRK2.5 were exclusively localized in the cytoplasm (Fig. 5F).

### Interactions between TmSnRK2s and TmbZIP1

**Fig. 6:**
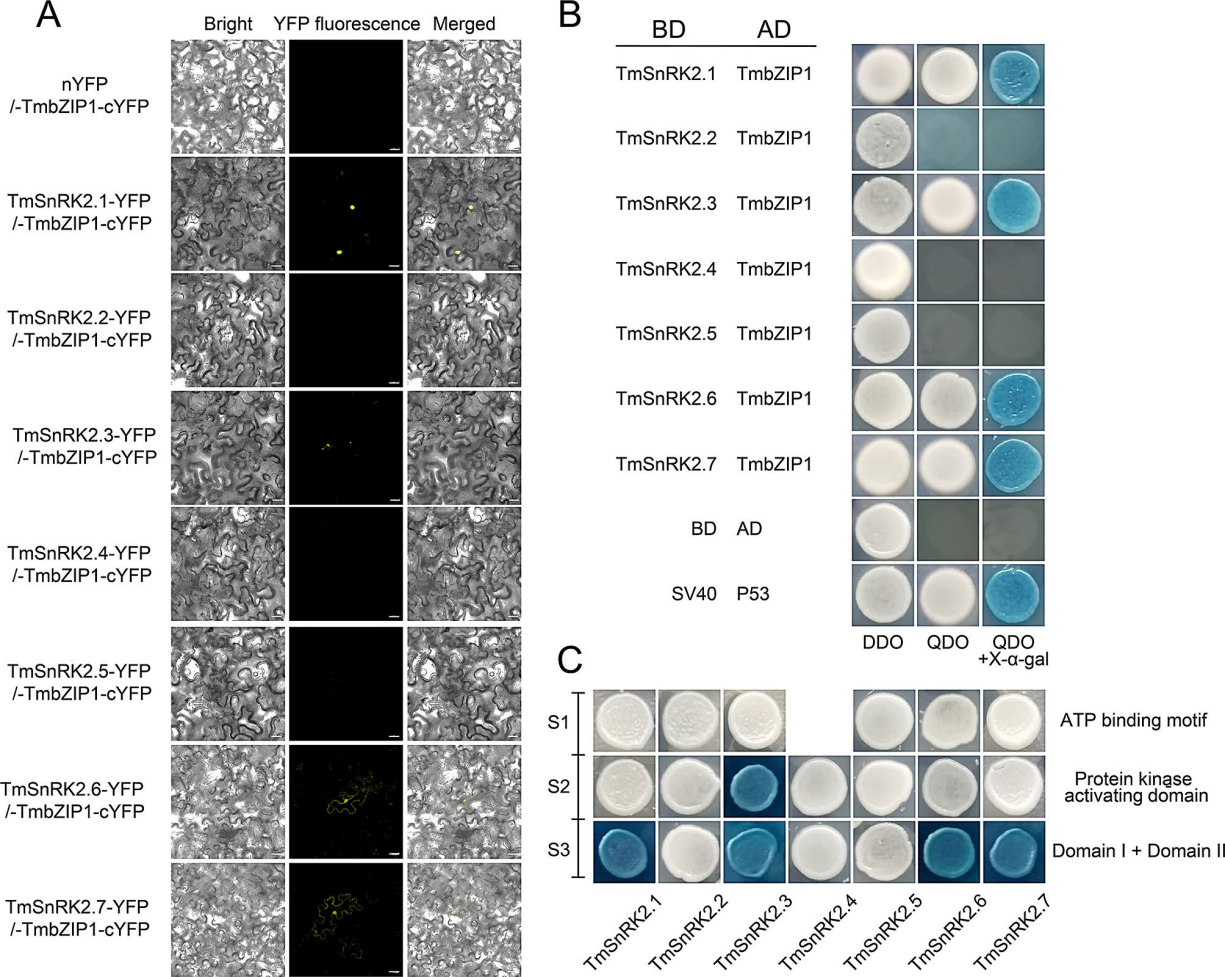
Interactions between TmSnRK2s and TmbZIP1 proteins

Previous research has demonstrated that TmSnRK2.6 interacts with TmbZIP1 [20]. To investigate whether other TmSnRK2 members also bind to TmbZIP1, we conducted Y2H and BiFC experiments. The results indicated that TmSnRK2.1, TmSnRK2.3, and TmSnRK2.7 can form heterodimers with TmbZIP1 in both yeast and tobacco (Fig. 6A and B). To further delineate the binding regions of TmSnRK2s to TmbZIP1, we prepared three segments of TmSnRK2s for segmentation experiments: the first segment (S1) encompasses the ATG-binding region, the second segment (S2) includes the protein kinase-activating domain, and the third segment (S3) comprises Domain I and Domain II (Fig. 6C). Additionally, the Y2H segmentation results revealed that TmSnRK2s bind to the TmbZIP1 segment containing Domains I and II. Based on the differences in amino acid sequences, we identified the motifs ‘Q(S/G)(V/D)(D/E)(I/L)××I(I/V)×EA’ and ‘D×(D/ED××D)’ as the core sites responsible for TmSnRK2s’ binding to TmbZIP1. Furthermore, we found that the protein kinase-activating domain of TmSnRK2.3 also interacts with TmbZIP1 (Fig. 6C). Further experiments will require phosphorylation detection through isotopic labeling and Phos-tag western blotting to elucidate the detailed functions [34, 35].

A, Y2H analysis was conducted to investigate the interaction between the TmbZIP1 and TmSnRK2s proteins. TmbZIP1 was fused to the pGADT7 vector, while the SnRK2s proteins were fused to the pGBKT7 vector; B, BiFC analysis was conducted to investigate the interaction between TmbZIP1 and SnRK2s proteins in tobacco cells. TmbZIP1 was fused to the C-terminal fragment of yellow fluorescent protein (YFP) (TmbZIP1-cYFP), and the TmSnRK2 proteins were linked to the N-terminus of a YFP fragment (SnRK2-nYFP). This analysis was repeated three times with six clones; C, The full-length TmSnRK2 proteins were divided into two or three segments to construct BD-SnRK2s-S1, BD-SnRK2s-S2, and BD-SnRK2s-S3 vectors, respectively. TmSnRK2.4 has no ATP-binding motif.

### The stability of TmbZIP1-Tm4CL1 promoter is affected by TmSnRK2s

**Fig. 7:**
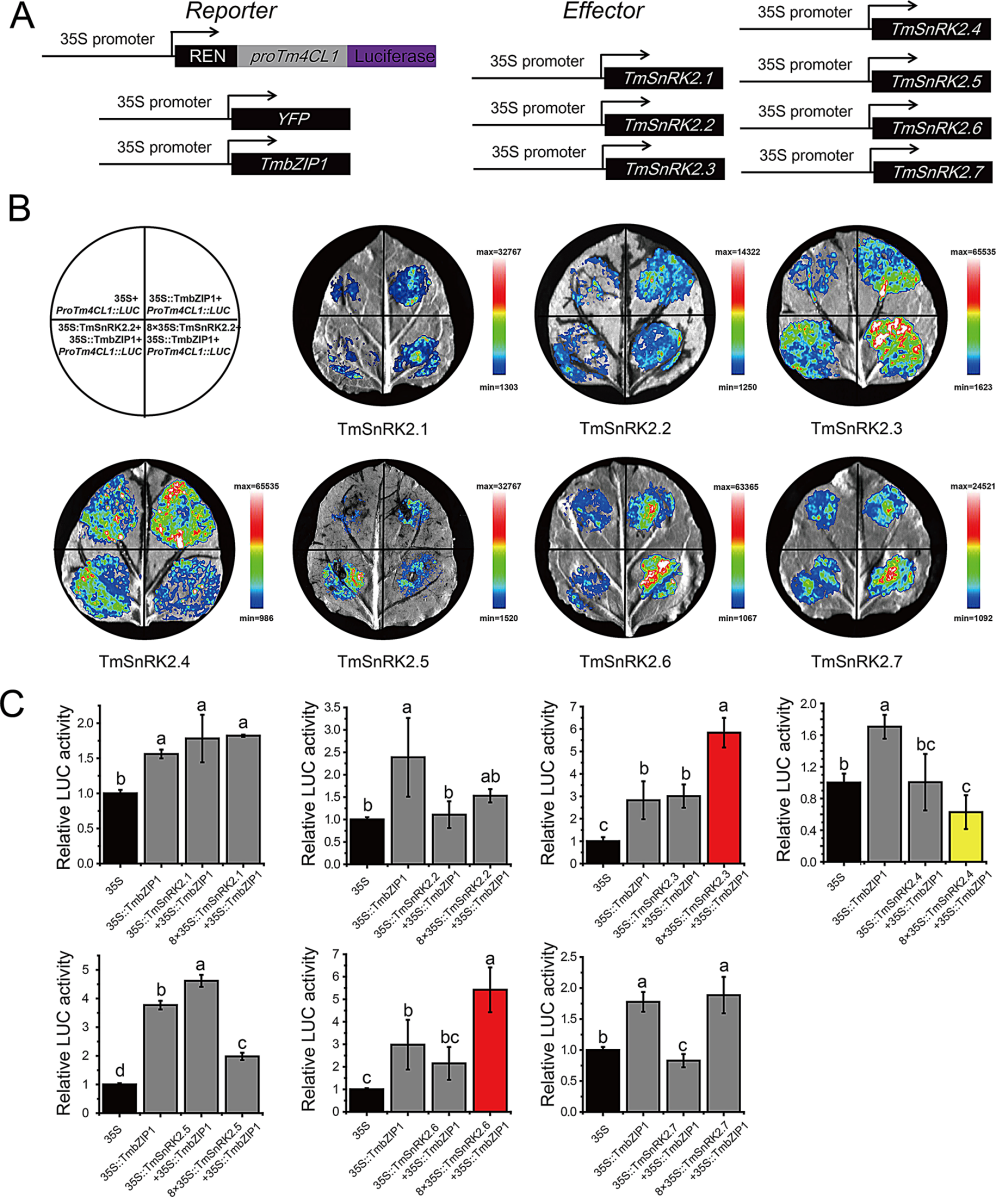
The SnRK2 proteins influence the stability of TmbZIP1 binding to the Tm4CL1 gene promoter

We have carried on a modified Dual-LUC assays to valuate the functions of TmSnRK2s in interacting with the TmbZIP1-Tm4CL1 model in *vivo*. Here, we added the constructed recombinant *Agrobacterium* GV3101 containing pHellsgate8-TmSnRK2s-GFP vectors to the previously reported Dual-LUC assay action system (TmbZIP1-Tm4CL1 as a positive control) in tobacco leaves [20]. The modified action system is shown in Fig. 7A, using pHellsgate8-TmSnRK2s as the dependent variable. The results were evaluated according to the LUC/REN ratio [36]. As shown in Fig. 7B and C, the ratio in the positive control (35 S: TmbZIP1) was significantly higher than that in empty control group (35 S). Those results of LUC enzyme activity indicated that TmSnRK2.3/2.6 significantly increased the TmbZIP1-Tm4CL1 expression, whereas TmSnRK2.2/2.5 showed no significant difference with positive control. However, the TmSnRK2.4 group showed inhibition of TmbZIP1-Tm4CL1 LUC activity whereas TmSnRK2.1 showed a decrease at low concentrations but an increase at high concentrations compared with the positive control. Together, these results indicate that TmSnRK2s can directly or indirectly affect the stability of the TmbZIP1-Tm4CL1 interaction.

A, Schematic representation of the luciferase (Luc) reporter vector incorporating the promoters of proTm4CL1, alongside the effector vector containing TmSnRK2s and TmbZIP1; B, Transient expression assays demonstrate that the co-expression of TmSnRK2s and TmbZIP1 proteins impacts the expression stability of the Tm4CL1 gene, representative images of *N. benthamiana* leaves, taken 72 h post-infiltration, are presented; C, Relative luciferase (LUC) activity from the transient expression analysis of the Tm4CL1 gene promoter co-infiltrated with a plasmid encoding TmbZIP1 fused to the 35 S promoter, six replicates were conducted for each experimental group, different letters denote statistical significance (*p* < 0.05). The columns marked in red indicate that TmSnRK2.3 and TmSnRK2.6 enhanced the TmbZIP1-Tm4CL1 interaction, while the column marked in yellow indicates that TmSnRK2.4 represses this interaction.

## Discussion

Chicoric acid is an important secondary metabolite in *T. mongolicum*; however, the chicoric acid biosynthesis pathway remains unclear. LC-MS was used for the detection of chicoric acid and its related compounds. Unlike untargeted metabolome results previously reported in ABA-treated *T. mongolicum* plants [20], a total of 11 chicoric acids and their related compounds were found in different tissues of *T. mongolicum*. The LC-MS data showed that chicoric acid concentrations were significantly higher in the leaves than in the roots and flowers (Table 1). However, ABA-induced transcriptome data were obtained from 1-month-old young *T. mongolicum* plants, whereas this study used 6-month-old mature plants to detect a higher number of chicoric acids and related compounds [20]. Based on these results, the differential transcriptomes were used to obtain detailed transcriptomic data. The length and diversity of unigenes in the tissue differential transcriptome of 6-month-old mature *T. mongolicum* were significantly higher than those in one-month-old seedlings treated with ABA (Fig. 2 and Table S3). Hence, the LC-MS and tissue differential transcriptome data provided in this study have laid the foundation for studying the secondary metabolites accumulation in different dandelion tissues. In addition, the results indicated that the TmbZIP1-Tm4CL1 complex is still an important component of the transcriptional regulation of chicoric acid biosynthesis in different tissues, as reported previously [20]. However, bHLH, ERF, and MYB TF members were also identified in this study and require further evaluation [37, 38]. Except for chicoric acid and the related compounds, terpenoids (dandelion sterols), flavonoids (luteolin and luteolin), and polysaccharides are also present in dandelion [1, 16, 38]. Therefore, the transcriptome data obtained from this study can be used to further explore the biosynthetic pathways and accumulation of these secondary metabolites.

It was observed that *A. thaliana* and *O. sativa* each contain ten SnRK2 members, whereas *Vitis vinifera* L. has eight members, and *Glycine max* possesses twenty-two members [39–41]. In diploid plants, such as wheat and mung bean, SnRK2s exhibit a high degree of evolutionary conservation [42, 43]. In this study, we systematically cloned and analyzed seven TmSnRK2 members from *T. mongolicum*, highlighting significant differences in the number of SnRK2s across various species. *T. mongolicum* is a triploid plant that differs from other *Taraxacum* species, such as *Taraxacum kok-saghyz* and *Taraxacum officinale* [2, 4]. This quantitative difference may be attributed to gene duplication, deletion, and polyploidy during their evolution. Generally, the number of gene family members in diploid and triploid species is lower compared to tetraploid, hexaploid, and octoploid species [16, 44]. Gene structure analysis revealed that the seven TmSnRK2 genes possess nine introns, consistent with patterns observed in *Chenopodium quinoa* and *Hordeum vulgare* [28, 42]. However, in *T. mongolicum*, conserved motif analysis indicated that all TmSnRK2s contain the typical SnRK2 domain, with the exception of TmSnRK2.4, which possesses only the protein kinase-activating domains I and II. One possible explanation for this pattern is that gene duplication and diversification provide abundant primary genetic material during evolution, while genetic variation allows adaptation to changing environmental conditions under natural selection [42]. Our results suggest that the TmSnRK2 genes in *T. mongolicum* may undergo selection and evolve to acquire new functions.

The SnRK2 family plays a crucial role in the regulation of plant phosphorylation [26–28]. Consequently, the predictive functions of the promoters associated with these SnRK2s provide a foundation for studying gene function. In this study, the analysis of the upstream 2000 bp promoter sequences of TmSnRK2s revealed that ABA, MeJA, SA hormone signals, as well as light signal transduction, were the most correlated factors (Table 2). Thus, SnRK2s are not only core regulatory elements in ABA hormone signaling but also integral to other hormone signaling pathways, abiotic stress responses, and overall stress responses [43]. TmSnRK2.3 and TmSnRK2.6 were significantly influenced by ABA signaling and exhibited high expression levels in leaves, despite belonging to different subfamilies. Similar findings have been reported previously [42]. In *A. thaliana*, AtSnRK2.1, AtSnRK2.4, AtSnRK2.5, AtSnRK2.9, and AtSnRK2.10 belong to group I and are ABA-insensitive; AtSnRK2.7 and AtSnRK2.8 belong to group II and exhibit weak ABA sensitivity; whereas AtSnRK2.2, AtSnRK2.3, and AtSnRK2.6 belong to group III and display strong ABA sensitivity [27]. Our findings indicate that TmSnRK2.1 is classified in group I, TmSnRK2.2, TmSnRK2.3, TmSnRK2.4, and TmSnRK2.5 are categorized in group II, while TmSnRK2.6 and TmSnRK2.7 are placed in group III. All TmSnRK2s were significantly affected by exogenous ABA. Interestingly, TmSnRK2.7 and TmSnRK2.6 share the same structural domain and are closely related. We speculate that TmSnRK2.7 and TmSnRK2.6 are homologous genes, despite significant differences in their promoters. For instance, both TmSnRK2.7 and TmSnRK2.6 contain one abscisic acid response element (ABRE) motif, whereas TmSnRK2.7 possesses three MeJA motifs but lacks three cis-acting elements, such as the TCA element, MBS, and P-BOX, along with an additional G-box motif. Therefore, we propose that the lack of significant differences in ABA hormone signaling for TmSnRK2.7 may be closely related to the number and structure of these cis-acting elements in its promoter.

In a recent study, it was found that ABA significantly enhances the biosynthesis of chicoric acid via the TmbZIP1-Tm4CL1 pathway [20]. Results from Y2H and BiFC assays demonstrated both direct and indirect regulatory relationships between TmSnRK2s and the TmbZIP1-Tm4CL1 complex (Fig. 6). Segment Y2H assays revealed that TmSnRK2.1, TmSnRK2.3, TmSnRK2.6, and TmSnRK2.7 bind directly to TmbZIP1 through S3 (comprising Domain I and Domain II), whereas TmSnRK2.3 interacts with TmbZIP1 through the ‘protein kinase activating’ domain at S2. This interaction leads to the phosphorylation of TFs and alters their properties [27, 29, 31]. Dual-LUC assays indicated that TmSnRK2.3 and TmSnRK2.6 significantly enhance the interaction between TmbZIP1 and Tm4CL1, while TmSnRK2.4 appears to diminish this interaction (Fig. 7). The reduction in fluorescence intensity of the TmbZIP1-Tm4CL1 module by SnRK2.4 may not be due to a direct effect on TmbZIP1; rather, TmSnRK2.4 may regulate the TmbZIP1-Tm4CL1 interaction indirectly through the phosphorylation of other TFs, potentially involving feedback regulation [45]. Further functional analyses utilizing mutant knockout and overexpression lines are necessary to elucidate the role of SnRK2s [34, 36, 41].

Based on current research, we assert that members of the SnRK2 family, which are exclusively found in *T. mongolicum*, play a crucial role. Considerable work has been conducted on the function of SnRK2 proteins in ABA hormone signaling. However, the roles of SnRK2s in other hormonal signaling pathways, such as those involving MeJA and indole-3-acetic acid (IAA), as well as their involvement in brassinosteroid and GA signaling, remain unclear. Notably, TmSnRK2.3 and TmSnRK2.6 have been reported to exhibit extensive phosphorylation activity, capable of simultaneously phosphorylating multiple TFs including ABI5, AREB1, and HAT1. Recent studies further demonstrate that SlSnRK2.3 is involved in the fruit development and maturation of watermelons by modulating ABA accumulation, GA biosynthesis and signaling, and sugar accumulation pathways [46]. Therefore, we hypothesize that TmSnRK2s proteins in *T. mongolicum* may influence the accumulation of chicoric acid and balance the biosynthesis of other secondary metabolites.

## Methods

### Plant materials

Seeds of *T. mongolicum* were collected and sown in a medicinal botanical garden located at a latitude of 32°03′15.71″ N and a longitude of 118°50′1.13″ E. Six well-developed flowering plants of *T. mongolicum*, each six months old, were harvested and divided into three parts: leaf, root, and flower. The samples were washed three times with sterile water, dried using sterile absorbent paper, and then placed into sterile 10 mL centrifuge tubes. Following this, the samples were frozen in liquid nitrogen and stored at -80 °C in preparation for transcriptome sequencing analysis and qRT-PCR assays [38]. The *T. mongolicum* samples were treated with 100 µM ABA for durations of 0, 2, 4, 8, and 24 h before being stored at -80 °C for subsequent qRT-PCR experiments. *N. benthamiana* plants were cultivated in 10 × 10 cm pots and maintained in a plant culture room at 24 °C under a photoperiod of 16 h light and 8 h dark.

### LC-MS analysis of Chicoric acid and related compounds in T. mongolicum

All tissue samples were removed from refrigeration and dried in a low-temperature freeze-dryer for 48 h until a stable weight was achieved. To extract phenolic compounds from the roots, leaves, and flowers of *T. mongolicum*, 0.5 g of each tissue sample was incubated in 10 mL of 80% methanol in water for 45 min. Each extraction was then diluted ten-fold to a final volume of 10 mL and filtered through a 0.22 μm filter. Chromatographic detection was performed using an Agilent 1260 UPLC-DAD-6530 ESI-QTOF MS system (Agilent Technologies Co., Ltd., USA) equipped with an Agilent Zorbax SB-C18 column (4.6 mm × 100 mm, 1.8 μm, 600 bar). A 2 µL aliquot of each sample was injected. The mobile phases consisted of an aqueous phase (Phase A: 0.1% formic acid) and an organic phase (Phase B: methanol). The gradient elution conditions were as follows: at 0 min, 10% B; at 10 min, 20% B; at 20 min, 20% B; at 50 min, 50% B; at 70 min, 100% B; and at 85 min, 100% B. The flow rate was set at 0.3 mL/min, the column temperature was maintained at 35 °C, and the scanning wavelength was 254 nm. The mass spectrometer operated under the following conditions: ESI ion source; negative ion scanning mode (100–1700 m/z) for sample mass spectrum signal collection; nebulizer pressure of 50 psi; drying gas flow of 10 mL/min; drying gas temperature of 350 °C; capillary voltage of 3500 V; and fragmentor voltage settings of 195 V (0–5 min), 135 V (5–60 min), and 175 V (60–85 min). Chemical components were identified by comparison with the Mass Hunter standard spectrum library [14, 47].

### RNA sequencing assembly and functional annotation analysis

Three different tissue samples (leaf, root, and flower) were retrieved from a -80 °C freezer for RNA extraction, which was conducted using the MiniBEST Plant RNA Extraction Kit (Code No. 9769) with three biological replicates. The final RNA concentration was standardized across samples prior to the construction of nine RNA libraries, designated as Leaf_1, Leaf_2, Leaf_3, Root_1, Root_2, Root_3, Flower_1, Flower_2, and Flower_3. Transcriptome sequencing was performed utilizing the Illumina HiSeq 4000 System, and data analysis was conducted using the online Majorbio Cloud Platform (www.majorbio.com) [20]. Clean reads were generated from the quality-controlled raw data of the sequenced *T. mongolicum* transcriptome using the online Sickle and SeqPrep tools. The raw data were assembled with the Trinity software (https://github.com/trinityrnaseq/trinityrnaseq) using the *T. mongolicum* genome (GWH; https://ngdc.cncb.ac.cn/search/) as a reference, yielding unigene sequences [48]. For functional annotation, unigenes were subjected to BLASTX alignment (E-value ≥ 10^− 5^). The Non-redundant Protein database (NR), Protein family database (Pfam), and Swiss-Prot database were employed to eliminate redundantly assembled proteins. Additionally, the Clusters of Orthologous Groups (COG), Gene Ontology (GO), and Kyoto Encyclopedia of Genes and Genomes (KEGG) databases were utilized for gene prediction and classification.

### Isolation, identification and characteristics of the TmSnRK2s

Hidden Markov Model (HMM) files corresponding to the SnRK2 domains associated with STKc_SnRK2-3 (specific SnRK2 kinase domain: PF00069) were obtained from the Pfam database. These HMM files were utilized in the online HMMER software (version 3.0) to perform a comparative search and identify the SnRK2 protein family within the annotated *T. mongolicum* genome (E-value < 10^− 5^). Additionally, nine SnRK2 protein sequences from *T. mongolicum* were analyzed using a local BLASTP search system, through which the protein sequences were further identified (E-value < 10^− 5^). We also examined all sequences in the abscisic acid (ABA) hormone-induced temporal transcriptome [20] and the tissue differential transcriptome (this study). Conserved TmSnRK2 domains were determined using the NCBI Conserved Domain Database (CDD) (https://www.ncbi.nlm.nih.gov/cdd/), Pfam, and SMART (http://smart.embl.de/) online tools. By integrating these methods, seven TmSnRK2s were cloned based on the unigene sequences provided by the *T. mongolicum* genome and transcriptome data. The primers used are listed in Supplementary Table S1. *T. mongolicum* DNA was extracted using the TaKaRa MiniBEST Plant Genomic DNA Extraction Kit (Code No. 9768), following the manufacturer’s instructions. All seven SnRK2s were cloned multiple times using *T. mongolicum* DNA and RNA as templates for comparative analyses (Table S2).

### Phylogenetic, gene structure and related information analyses

All TmSnRK2 protein sequences were aligned using IQ-TREE with ModelFinder Plus (MFP) to identify the most suitable model for tree building analysis. This process simultaneously generated the Akaike Information Criterion (AIC) and the Bayesian Information Criterion (BIC). The ‘contree’ format annotation files were utilized with the iTOL online analysis tool to construct the phylogenetic tree, while GeneDoc software was employed for amino acid alignment. The TmSnRK2 genomic sequences were imported into TBtools to create exon-intron structural diagrams, and MEME (http://meme-suite.org/tools/meme) was used to analyze conserved protein motifs. The promoters of the TmSnRK2 genes, extending 2000 base pairs upstream of the ATG start codon, were extracted using TBtools, and the cis-elements within these promoter regions were analyzed using the online PlantCARE tool.

### Expression patterns analysis of TmSnRK2s

Transcriptome data from various tissues of *T. mongolicum*, including roots, leaves, and flowers (accession number PRJNA861012), as well as data from ABA-induced treatments (accession number PRJNA860343 [20]),, were obtained from the NCBI BioProject database. qRT-PCR was conducted using PowerTrack SYBR Green (Thermo Fisher Scientific, USA) to quantify gene expression levels, with β-actin and Glyceraldehyde-3-phosphate Dehydrogenase (GAPDH) serving as the reference genes. Relative expression levels were calculated using the 2^−ΔΔCt^ method, as previously reported [38]. The qRT-PCR primers were designed using the Primer 3 (v. 4.0) online tool (see Table S1).

### Subcellular localization of TmSnRK2s

The complete coding sequences of TmSnRK2s were individually cloned into the pHellsgate8-GFP vector and subsequently sequenced by Shanghai Sangon Biological Co., Ltd. (Shanghai, China). The recombinant vectors, designated as pHellsgate8-TmSnRK2s-GFP, were transferred into *Agrobacterium* GV3101 using the freeze-thaw method. The strains containing the recombinant vectors were identified by PCR, employing 35SF and G1-R primers (Table S1). *Agrobacteria* harboring pHellsgate8-TmSnRK2s-GFP and the empty vector were initially cultured overnight in a 2 mL sterile centrifuge tube and subsequently transferred to a 100 mL sterile triangular flask, where they were cultured until reaching OD_600_ = 0.8–1.0. The *Agrobacteria* suspension was centrifuged, and the pellet was resuspended in a resuspension medium consisting of 10 mM MgCl_2_ and 100 µM acetosyringone. Following a 3 h incubation in the dark, the backs of well-grown, 2-month-old tobacco leaves were injected with the *Agrobacteria* and incubated in the dark for 48 to 72 h. The leaves were then examined using a confocal microscope (Zeiss LSM 900, Germany). The experimental methodology has been previously described [20].

### Y2H assays

The full-length coding sequences of TmSnRK2s were individually cloned into the pGBKT7-rec vector, while TmbZIP1 was cloned into the pGADT7-rec vector. Segments of TmSnRK2s (S1, S2, and S3) were subsequently inserted into the pGBKT7-rec plasmid to yield pGBKT7-TmSnRK2s-S1/S2/S3. Recombinant plasmids (pGBKT7-TmSnRK2s + pGADT7-TmbZIP1) were co-transformed into yeast using the freeze-thaw method, and their presence was confirmed through PCR assays [20, 49]. To assess the interaction between TmSnRK2s and TmbZIP1, SD/-Trp/-Leu (DDO) solid medium was employed for the initial screening of positive strains. Subsequently, SD/-Trp/-Leu/-His/-Ade (QDO) medium was utilized for re-screening, and the identified strains were transferred to SD/Trp/Leu/-His/-Ade/+x-α-gal (QDO + x-α-gal) for blue-white screening based on the β-galactosidase color reaction. The controls included pGBKT7-SV40 + pGADT7-p53 as the positive control, pGBKT7-rec + pGADT7-TmbZIP1 as the negative control, and pGBKT7-rec + pGADT7-rec as the blank control. Six individual yeast colonies were analyzed as replicates.

### BiFC assays

To investigate the interaction between TmSnRK2s and TmbZIP1, BiFC assays were conducted in tobacco. TmSnRK2s were cloned into the pXY106-nYFP vector, while TmbZIP1 was cloned into the pXY104-cYFP vector through homologous recombination. The recombinant nYFP and cYFP vectors were then transformed into *Agrobacterium* GV3101 strains, which were subsequently verified by PCR using the primers 104 F + TmSnRK2sR and TmbZIP1F + 106R (see Table S1). *Agrobacterium* cultures containing nYFP and cYFP vectors were grown to the same optical density and mixed in equal volumes for injection into the backs of tobacco leaves. The mixture of *Agrobacterium* GV3101 containing nYFP and TmbZIP1-cYFP served as a negative control. BiFC assays were performed as previously described [32, 50].

### Dual-LUC assays

We observed that TmbZIP1 directly interacts with proTm4CL1 in tobacco [20, 49]. Modified Dual-LUC assays were conducted to investigate the stability of the TmbZIP1-proTm4CL1 complex as influenced by TmSnRK2s. The recombinant *Agrobacterium* GV3101, which harbored the pHellsgate8-TmSnRK2s-GFP construct, was utilized as the effector. In contrast, a mixture of *Agrobacterium* containing pGreen0800-proTm4CL1 and pHellsgate8-TmbZIP1-GFP served as the combined reporter. The dual-luciferase assays were performed six times to ensure reproducibility.

## Electronic supplementary material

Below is the link to the electronic supplementary material.

Supplementary Material 1: Figure S1. TIC chromatograms of root, leaf, and flower analyzed on LC/MS, Figure S2. Species distribution, E-value distribution and similarity distribution of transcriptome analysis data, Figure S3. COG classification chart. The ABSCISSA represents the functional classification of COG (expressed in Majuscule A-Z) and the ordinate represents the number of unigenes with such a function, Figure S4. Principal component analysis of *T. mongolicum* transcriptome in different tissues, Figure S5. Volcano plot of Root/Leaf, Root/Flower, and Leaf/Flower groups, with the red color indicating upregulated unigenes and the blue color indicating downregulated unigenes, Figure S6. KEGG enrichment analysis of comparative transcriptome profiling data, Figure S7. Phylogenetic trees construction.

Supplementary Material 2: Table S1. Primers used in this study, Table S2. Summary of transcriptomes from three tissues (flower, root, leaf) *T. mongolicum* samples, Table S3. Summary of the sequence assembly results, Table S4. Statistics of annotations for assembled unigenes in six public databases, Table S5. Promoters and CDS sequences of TmSnRK2s in *T. mongolicum*, Table S6. Protein sequence used of AtSnRK2s and OsSAPKs and TmSnRK2 in *T. mongolicum*, Table S7. Raw data of tissue and ABA treatment qRT-PCR results.

## Data Availability

*Taraxacum mongolicum* RNA-seq data from three tissues were obtained from the Sequence Read Archive (SRA; accession number PRJNA861012) of NCBI.

## Abbreviations

4CL: 4-Coumarate coenzyme A ligase
BiFC: Bimolecular fluorescence complementation
C4H: Cinnamate 4-hydroxylase
Dual-LUC: Dual-Luciferase reporter Assay
HPLC: High-performance liquid chromatography
HQT: Hydroxycinnamoyl-CoA quinyl hydroxycinnamyltransferase
HTT: Hydroxycinnamoyl tartrate
LC-MS: Liquid chromatograph mass spectrometer
LUC/REN: Luciferin/Renilla ratio
ORF: Open reading frame
PAL: Phenylalanine ammonia lyase
qRT-PCR: Quantitative real-time polymerase chain reaction
SCPL: Serine carboxypeptidase-like proteins
TCM: Tradtitonal Chinese medicine
SnRK: Sucrose non-fermenting 1-related protein kinases
TF: Transcription factor
PM: Transcripts per million
Y2H: Yeast two-hybrid assay
