# Supplementary material for: Identification and characterization of the TmSnRK2 family proteins related to chicoric acid biosynthesis in Taraxacum mongolicum
Source: BMC Genomics. 2025 Mar 20;26:276. doi: 10.1186/s12864-025-11460-w (PMC11927344; doi:10.1186/s12864-025-11460-w)
Supplement: Supplementary file 1 — Supplementary Material 1: Figure S1. TIC chromatograms of root, leaf, and flower analyzed on LC/MS, Figure S2. Species distribution, E-value distribution and similarity distribution of transcriptome analysis data, Figure S3. COG classification chart. The ABSCISSA represents the functional classification of COG (expressed in Majuscule A-Z) and the ordinate represents the number of unigenes with such a function, Figure S4. Principal component analysis of T. mongolicum transcriptome in different tissues, Figure S5. Volcano plot of Root/Leaf, Root/Flower, and Leaf/Flower groups, with the red color indicating upregulated unigenes and the blue color indicating downregulated unigenes, Figure S6. KEGG enrichment analysis of comparative transcriptome profiling data, Figure S7. Phylogenetic trees construction. [file 12864_2025_11460_MOESM1_ESM.docx]

**Figure S1-1.** TIC chromatograms of root analyzed on LC/MS. 1-11, 5-O-Caffeoylshikimic acid, tartaric acid, Quinic acid, L-Phenylalanine, Caftaric acid, *p*-Coumalic acid, Caffeic acid, Chlorogenic acid, L-cichoric acid, D-cichoric acid, and Isochlorogenic acid A.

**Figure S1-2.** TIC chromatograms of leaf analyzed on LC/MS. 1-11, 5-O-Caffeoylshikimic acid, tartaric acid, Quinic acid, L-Phenylalanine, Caftaric acid, *p*-Coumalic acid, Caffeic acid, Chlorogenic acid, L-cichoric acid, D-cichoric acid, and Isochlorogenic acid A.

**Figure S1-3.** TIC chromatograms of flower analyzed on LC/MS. 1-11, 5-O-Caffeoylshikimic acid, tartaric acid, Quinic acid, L-Phenylalanine, Caftaric acid, *p*-Coumalic acid, Caffeic acid, Chlorogenic acid, L-cichoric acid, D-cichoric acid, and Isochlorogenic acid A.


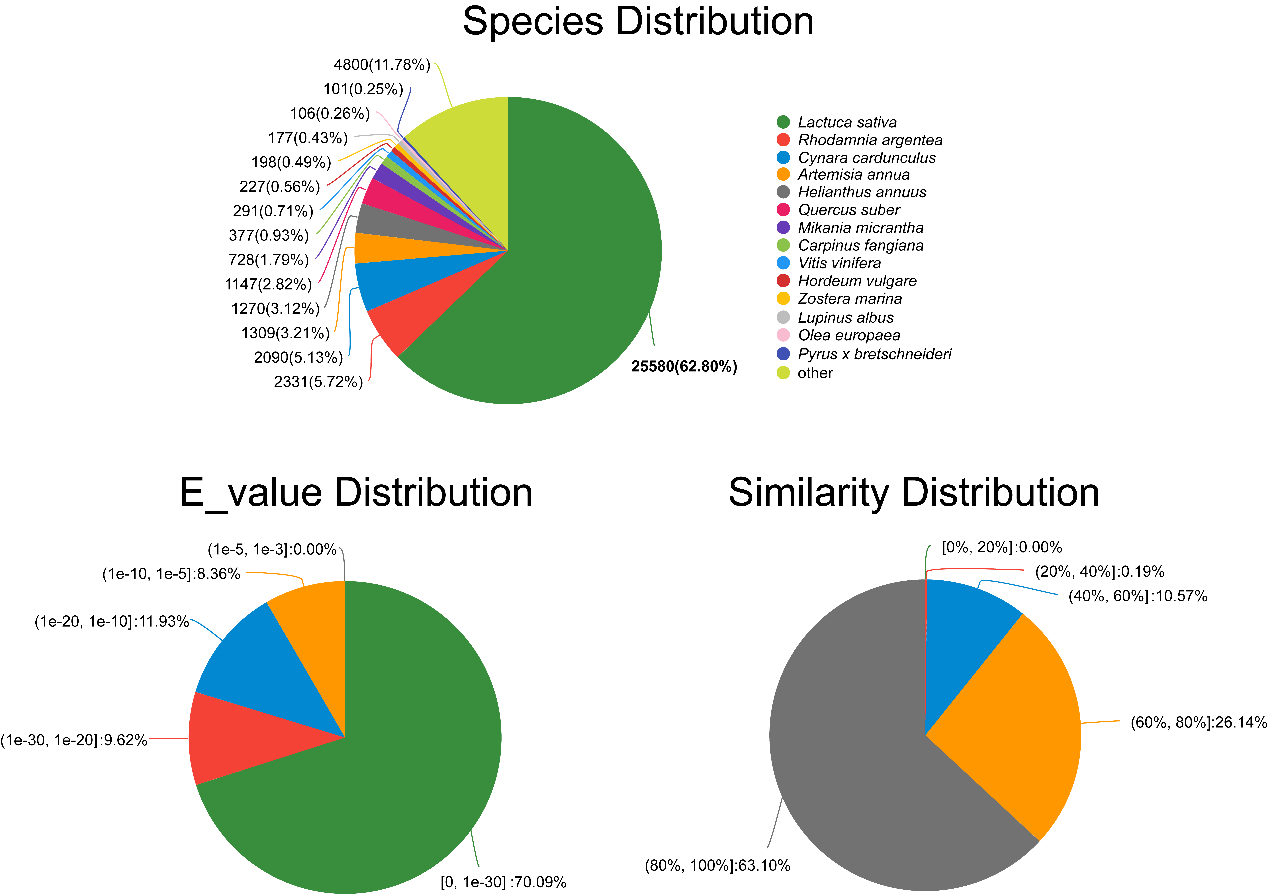


**Figure S2.** Species distribution, E-value distribution and similarity distribution of transcriptome analysis data. A, Distribution of NR annotated species. The fan chart showed the species names of NR on *T.* *mongolicum*. The number indicated the number of unigenes of *T.* *mongolicum* on the comparison of different species, and the percentage in brackets indicated the proportion of unigenes of *T.* *mongolicum* on the comparison of different species. B, Each sector represented a similarity interval. The larger sector area, the more unigenes in this similarity interval. C, Each sector represented an e-value interval. The smaller the e-value, the higher the reliability of the matching results. The larger sector area, the more unigenes with e-value in this interval.


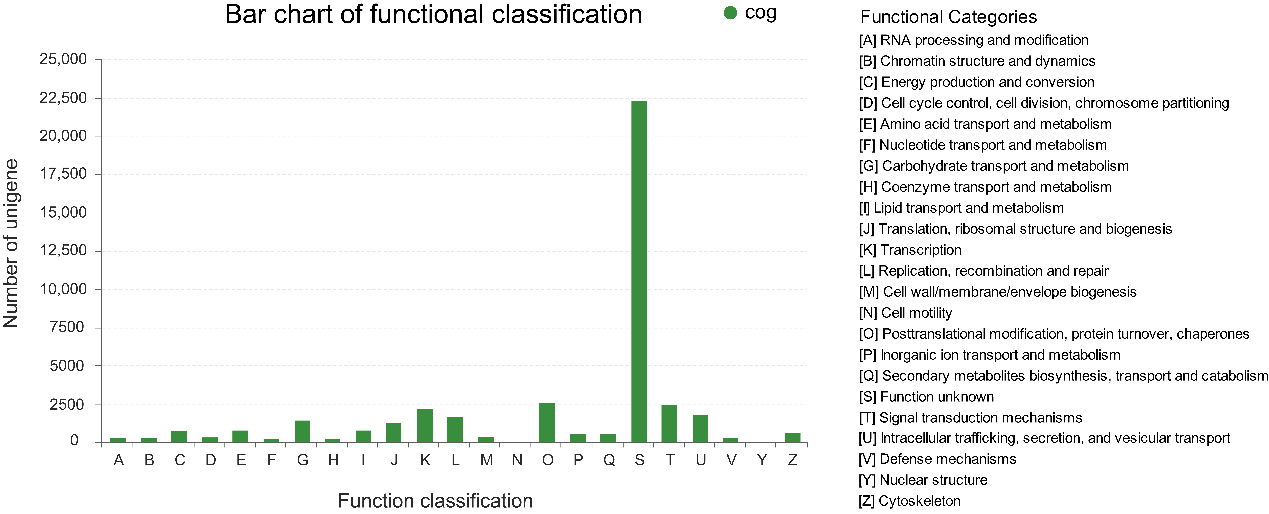


**Figure S3.** COG classification chart. The ABSCISSA represents the functional classification of COG (expressed in Majuscule A-Z) and the ordinate represents the number of unigenes with such a function.


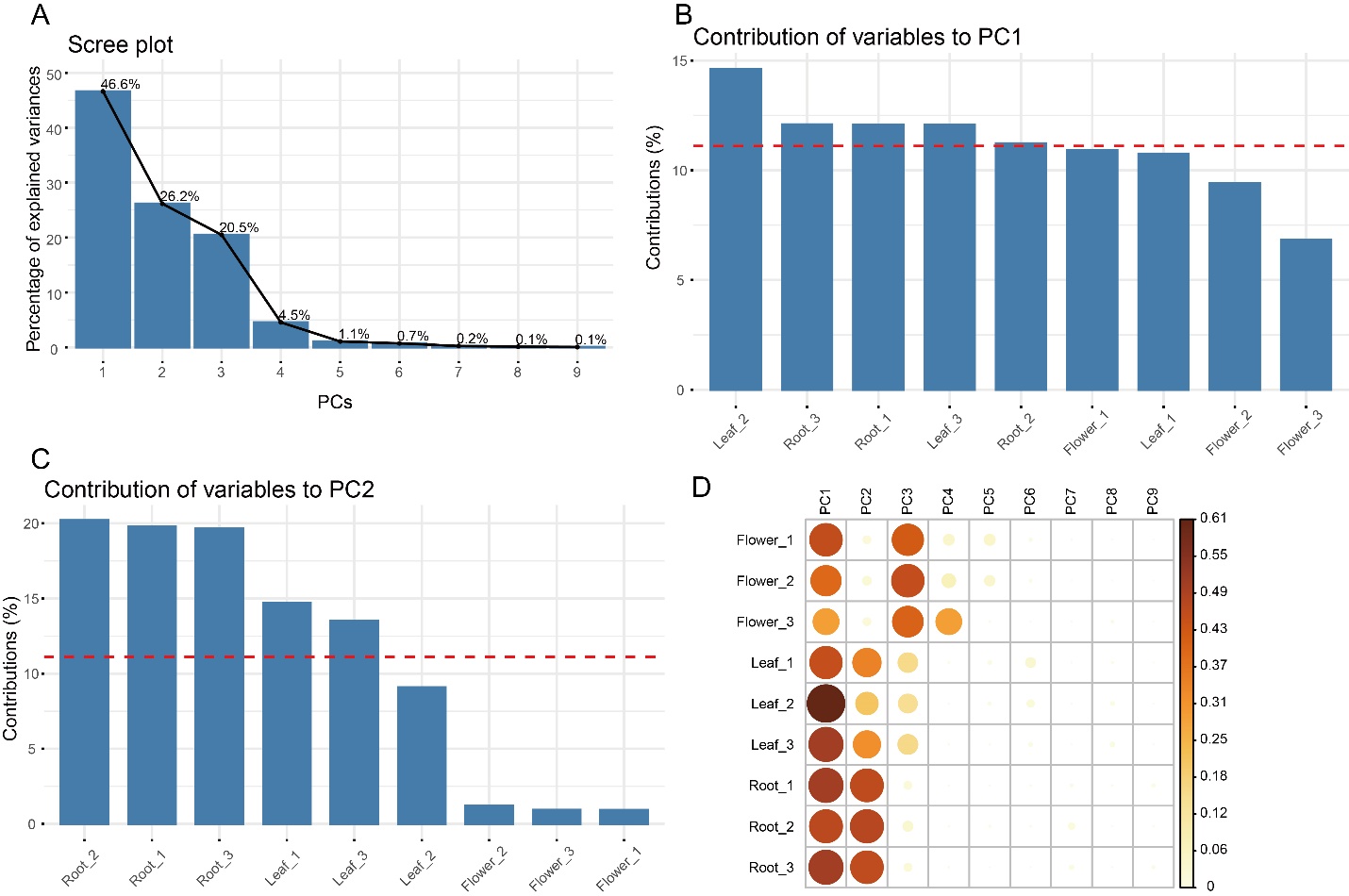


**Figure S4**. Principal component analysis of *T. mongolicum* transcriptome in different tissues

A. The scree plot illustrates the percentage of variance explained by different principal components (PCs). The graph indicates that the principal component (PC1-PC5) accounts for approximately 46.6%, 26.2%. 20.5%, 4.5%, and 1.1% for each. The variance explained by subsequent principal components decreases progressively. B. The column chart depicting the contribution of variables to PC1 highlights the significance of each variable in relation to the first principal component. The figure shows that variables such as "Root_1," "Root_2," "Root_3," "Leaf_1," "Leaf_2," and "Flower_1" make substantial contributions to PC1. The contribution of each variable is represented by a blue bar chart, with values indicated on the y-axis. C. The column chart illustrating the contribution of variables to PC2 reveals the significance of each variable with respect to the second principal component. The figure indicates that variables such as "Root_1," "Root_2," "Root_3," and "Leaf_1," "Leaf_2" contribute significantly to PC2. Similar to PC1, the contribution of each variable is depicted by a blue bar chart, with values shown on the y-axis. D. The correlation matrix displays the relationships among all variables.


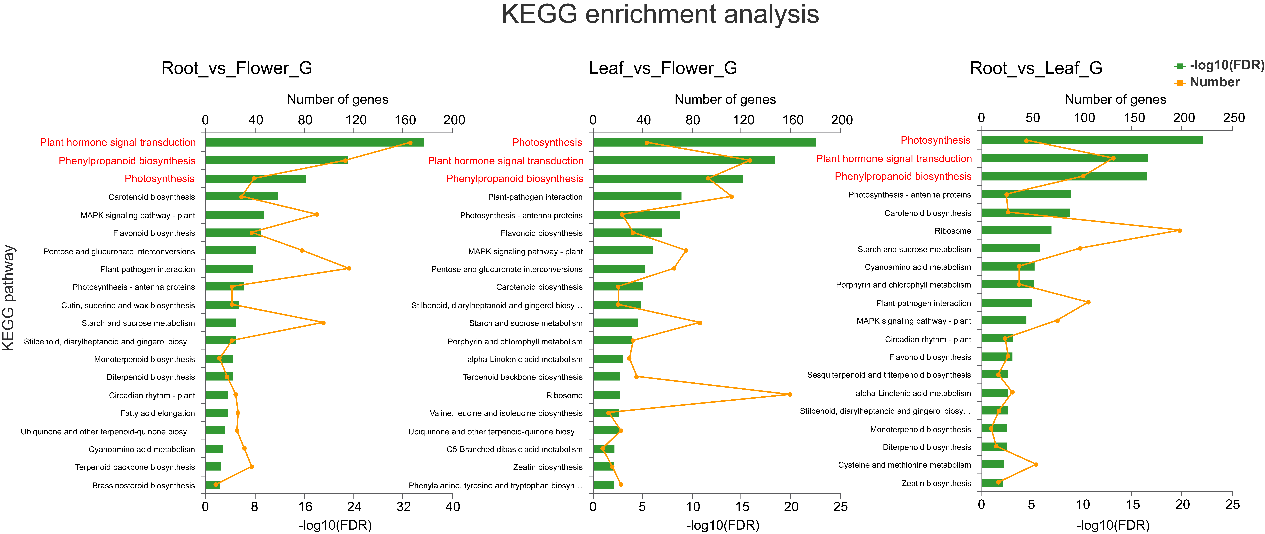


**Figure S5. KEGG enrichment analysis of comparative transcriptome profiling data.** The ordinate represented KEGG pathway, and the upper abscissa represented the number of unigenes compared to this pathway, corresponding to different points on the broken line. The lower abscissa represented the significance level of enrichment, corresponding to the height of the column.


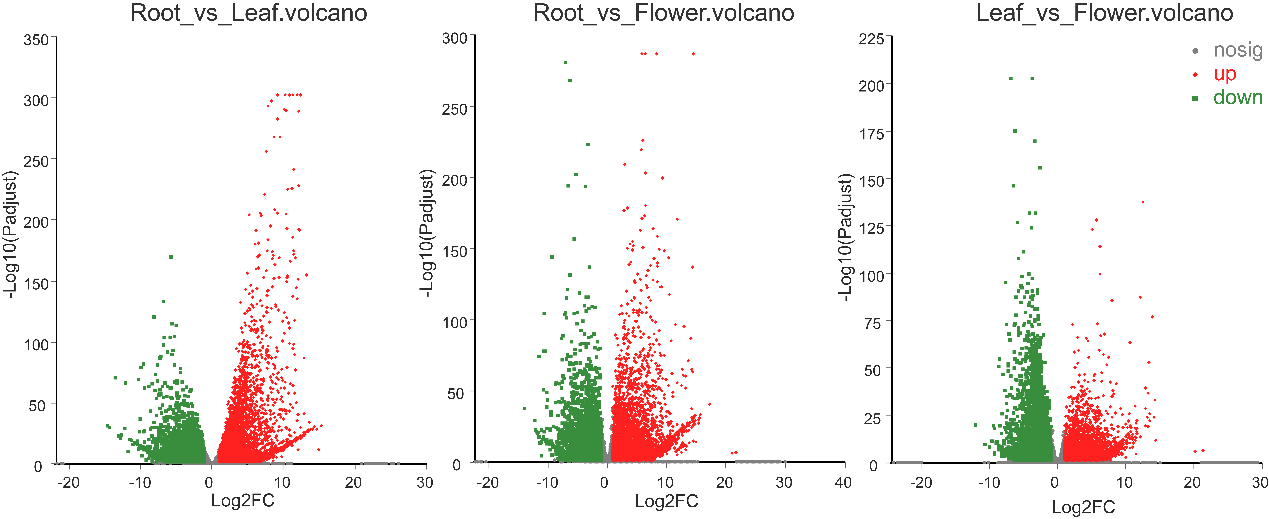


**Figure S6.** Volcano plot of Root/Leaf, Root/Flower, and Leaf/Flower groups, with the red color indicating upregulated unigenes and the blue color indicating downregulated unigenes.


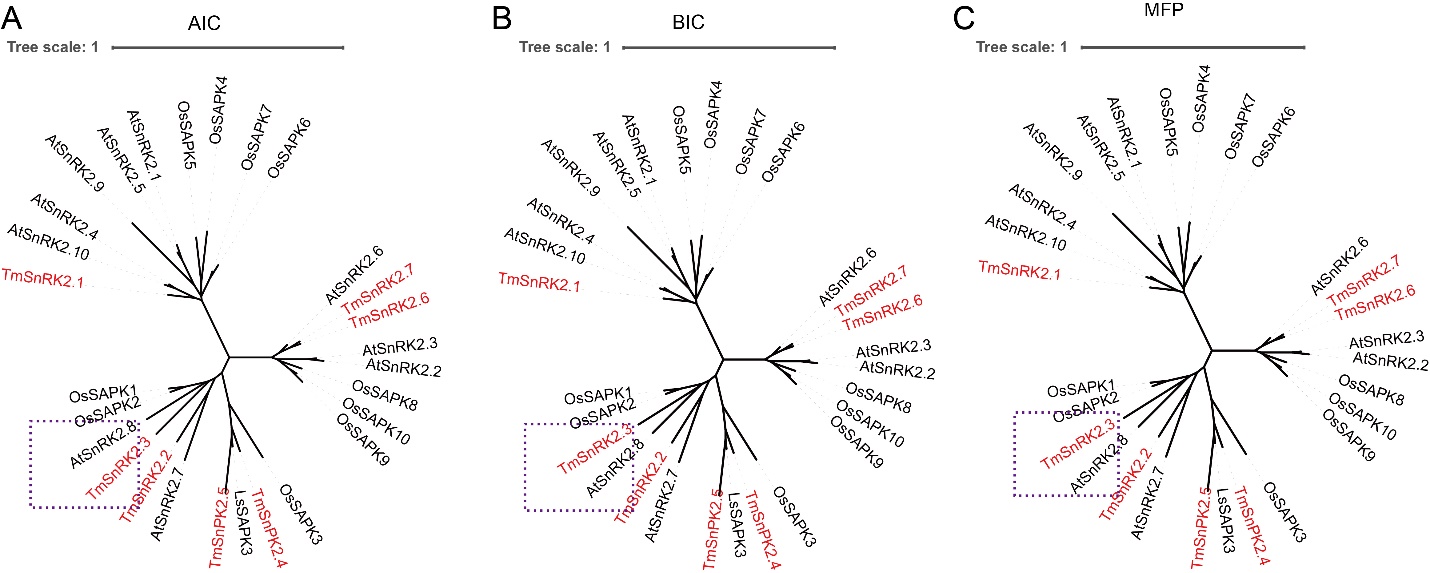


**Figure S7.** Phylogenetic trees construction. AIC, BIC and MFP The IQ-TREE software was utilized to construct phylogenetic trees for the SnRK2 family members in *T. mongolicum*, *A. thaliana* and *O. sativa L*. The trees were generated by selecting the optimal model based on Akaike Information Criterion (AIC) and Bayesian Information Criterion (BIC) analyses. The results indicated minor differences among the trees (see purple box).
