# Supplementary material for: Identification and characterization of the TmSnRK2 family proteins related to chicoric acid biosynthesis in Taraxacum mongolicum
Source: BMC Genomics. 2025 Mar 20;26:276. doi: 10.1186/s12864-025-11460-w (PMC11927344; doi:10.1186/s12864-025-11460-w)
Supplement: Supplementary file 2 — Supplementary Material 2: Table S1. Primers used in this study, Table S2. Summary of transcriptomes from three tissues (flower, root, leaf) T. mongolicum samples, Table S3. Summary of the sequence assembly results, Table S4. Statistics of annotations for assembled unigenes in six public databases, Table S5. Promoters and CDS sequences of TmSnRK2s in T. mongolicum, Table S6. Protein sequence used of AtSnRK2s and OsSAPKs and TmSnRK2 in T. mongolicum, Table S7. Raw data of tissue and ABA treatment qRT-PCR results. [file 12864_2025_11460_MOESM2_ESM.docx]

| **Supplementary Table S1.primers used in this study** | | |
| --- | --- | --- |
| **target** | **primer name** | **sequence (5'-3')** |
| qRT-PCR | qRT-SnRK2.1F | AGTTGCGAGGTTGATGAGGA |
|  | qRT-SnRK2.1R | GACAATAGCAAGGTGGGTGG |
|  | qRT-SnRK2.2F | CAGAGATCCAAATGCACCCG |
|  | qRT-SnRK2.2R | GGCGATCCATCACAAAGCAT |
|  | qRT-SnRK2.3F | CTTGATGGAAGTGCAGCTCC |
|  | qRT-SnRK2.3R | CCCCGACTAGCATCACGTAT |
|  | qRT-SnPK2.4F | TTCGCAACCAAAGTCGACTG |
|  | qRT-SAPK2.4R | ACTGAACGCTCATGATTCGC |
|  | qRT-SAPK2.5F | GAAGTGGCAAGGCTGGTTAG |
|  | qRT-SAPK2.5R | CTTCACTGAATCTGCCACGG |
|  | qRT-SnRK2.6F | GCCCAGCTCCTCGTTTAAAG |
|  | qRT-SnRK2.6R | TTTGGGTTCTTCGGGGTCTT |
|  | qRT-SnRK2.7F | ACGAAAACGCAATGGACCAA |
|  | qRT-SnRK2.7R | AATCTCCCCACTGCTGTCAA |
|  | qRT-bZIP1F | TTCAACCGCAAATCACAGAG |
|  | qRT-bZIP1R | TGGAGATCCAGCACCAAGAC |
|  | qRT-AREB1F | CAAACCCGCAACAACAACTT |
|  | qRT-AREB1R | CCCTCACAACTCCGCTAAAT |
|  | ACTIN R | AGCAGCTTCCATTCCGATCA |
|  | ACTIN F | GGTTACATGTTCACCACCAC |
| promoter clone | Pro-4CL1-F | ACATACTTCATCCTCAACGTAACC |
|  | Pro-4CL1-R | ATAATCTCCTTTTCCGGCTCCAT |
|  | Pro-bZIP1-F | CCTAAAGAGTATCTCTAGAAGG |
|  | Pro-bZIP1-R | CTGAAGTTGATAAACGAACTCAT |
|  | Pro-AREB1-F | GTTACGGATGCTATGAGCTAC |
|  | Pro-AREB1-R | GGTTTCCGAAGCTTTTGAAATTC |
| Subcellular localization and Dual-LUC(G1-GFP) | G1-SnRK2.1F | TTTGGAGAGGACACGCTCGAGATGGAGGAGAAGTACGAAC |
|  | G1-SnRK2.1R | GCTCACCATGAATTCCTCGAGCATTTGAGCAGAATAATCAATC |
|  | G1-SnRK2.2F | TTTGGAGAGGACACGCTCGAGATGGTCTTCCCTCCAAATC |
|  | G1-SnRK2.2R | GCTCACCATGAATTCCTCGAGTAATGCACAAACAAAATCC |
|  | G1-SnRK2.3F | TTTGGAGAGGACACGCTCGAGATGGAAAGATATGAACTTG |
|  | G1-SnRK2.3R | GCTCACCATGAATTCCTCGAGCAATGAGCAACCATAATCATC |
|  | G1-SnPK2.4F | TTTGGAGAGGACACGCTCGAGATGGAAATTTGTCACAGAG |
|  | G1-SAPK2.4R | GCTCACCATGAATTCCTCGAGACCTTGGCCACCATAATCTC |
|  | G1-SAPK2.5F | TTTGGAGAGGACACGCTCGAGATGGAGAAGTACGAGCTTG |
|  | G1-SAPK2.5R | GCTCACCATGAATTCCTCGAGAACAGGTCCAATTTCTCCAC |
|  | G1-SnRK2.6F | TTTGGAGAGGACACGCTCGAGATGGATCGATCGGCGCTTAC |
|  | G1-SnRK2.6R | GCTCACCATGAATTCCTCGAGTTATAACGCGTATACAATC |
|  | G1-SnRK2.7F | TTTGGAGAGGACACGCTCGAGATGGATCGATCTGCGCTTAC |
|  | G1-SnRK2.7R | GCTCACCATGAATTCCTCGAGTCACATTGCATAAACTATC |
|  | G1-AREB1F | TTTGGAGAGGACACGCTCGAGATGAACTTCAAAAGCTTCGG |
|  | G1-AREB1R | GCTCACCATGAATTCCTCGAGCCATGGCCCCGATTGTG |
|  | G1-bZIP1F | TTTGGAGAGGACACGCTCGAGATGAGTTCGTTTATCAACTTC |
|  | G1-bZIP1R | GCTCACCATGAATTCCTCGAGCCATGGCCCTGTTAATG |
| Dual-LUC(pGREEN-0800) | 0800-pro4CL1F | CAGCCCGGGGGATCCACATACTTCATCCTCAACGTAACC |
|  | 0800-pro4CL1R | TGGCGTCTTCCATGGATAATCTCCTTTTCCGGCTCCATC |
|  | 0800-probZIP1F | CAGCCCGGGGGATCCCCTAAAGAGTATCTCTAGAAGGACA |
|  | 0800-probZIP1R | TGGCGTCTTCCATGGCTGAAGTTGATAAACGAACTCAT |
|  | 0800-proAREB1F | CAGCCCGGGGGATCCGTTACGGATGCTATGAGCTAC |
|  | 0800-proAREB1R | TGGCGTCTTCCATGGAGTAGAAATTCTCTCAACTTTG |
| Y1H (ABRE-cis element ) | pLacZ-4CL1-1F | AATTCTGGCACGTGTCAATGGCACGTGTCAATGGCACGTGTCAAC |
|  | pLacZ-4CL1-1R | TCGAGTTGACACGTGCCATTGACACGTGCCATTGACACGTGCCAG |
|  | pLacZ-4CL1-2F | AATTCTTATCACGTCAACTTATCACGTCAACTTATCACGTCAACC |
|  | pLacZ-4CL1-2R | TCGAGGTTGACGTGATAAGTTGACGTGATAAGTTGACGTGATAAG |
|  | pLacZ-4CL1-3F | AATTCATTCCACGTTATTATTCCACGTTATTATTCCACGTTATTC |
|  | pLacZ-4CL1-3R | TCGAGAATAACGTGGAATAATAACGTGGAATAATAACGTGGAATG |
|  | pLacZ-bZIP1-1F | AATTCCTAAACGTGATTCTAAACGTGATTCTAAACGTGATTC |
|  | pLacZ-bZIP1-1R | TCGAGAATCACGTTTAGAATCACGTTTAGAATCACGTTTAGG |
|  | pLacZ-bZIP1-2F | AATTCAACTACGTGTCAAACTACGTGTCAAACTACGTGTCAC |
|  | pLacZ-bZIP1-2R | TCGAGTGACACGTAGTTTGACACGTAGTTTGACACGTAGTTG |
|  | pLacZ-bZIP1-3F | AATTCGCTGACGTGGAAGGCTGACGTGGAAGGCTGACGTGGAAGC |
|  | pLacZ-bZIP1-3R | TCGAGCTTCCACGTCAGCCTTCCACGTCAGCCTTCCACGTCAGCG |
|  | pLacZ-AREB1-1F | AATTCTTGAACGTTGAATTTGAACGTTGAATTTGAACGTTGAATC |
|  | pLacZ-AREB1-1R | TCGAGATTCAACGTTCAAATTCAACGTTCAAATTCAACGTTCAAG |
|  | pLacZ-AREB1-2F | AATTCCCAAACGTTCTCTCCAAACGTTCTCTCCAAACGTTCTCTC |
|  | pLacZ-AREB1-2R | TCGAGAGAGAACGTTTGGAGAGAACGTTTGGAGAGAACGTTTGGG |
|  | pLacZ-AREB1-3F | AATTCTTCTCACGTTATCTTCTCACGTTATCTTCTCACGTTATCC |
|  | pLacZ-AREB1-3R | TCGAGGATAACGTGAGAAGATAACGTGAGAAGATAACGTGAGAAG |
| Y1H (pB42AD ) | pB42AD-bZIP1F | GCCTCTCCCGAATTCATGAGTTCGTTTATCAACTTC |
|  | pB42AD-bZIP1R | GAGTCGGCCGAATTCCTACCATGGCCCTGTTAATG |
|  | pB42AD-AREB1F | GCCTCTCCCGAATTCATGAACTTCAAAAGCTTCGG |
|  | pB42AD-AREB1R | GAGTCGGCCGAATTCTCACCATGGCCCCGATTGTG |
| Y2H(BD) | BD-SnRK2.1F | ATGGCCATGGAGGCCGAATTCATGGAGGAGAAGTACGAAC |
|  | BD-SnRK2.1R | TCGACGGATCCCCGGGAATTCCATTTGAGCAGAATAATCAATC |
|  | BD-SnRK2.2F | ATGGCCATGGAGGCCGAATTCATGGTCTTCCCTCCAAATC |
|  | BD-SnRK2.2R | TCGACGGATCCCCGGGAATTCTAATGCACAAACAAAATCC |
|  | BD-SnRK2.3F | ATGGCCATGGAGGCCGAATTCATGGAAAGATATGAACTTG |
|  | BD-SnRK2.3R | TCGACGGATCCCCGGGAATTCCAATGAGCAACCATAATCATC |
|  | BD-SnPK2.4F | ATGGCCATGGAGGCCGAATTCATGGAAATTTGTCACAGAG |
|  | BD-SnPK2.4R | TCGACGGATCCCCGGGAATTCACCTTGGCCACCATAATCTC |
|  | BD-SnPK2.5F | ATGGCCATGGAGGCCGAATTCATGGAGAAGTACGAGCTTG |
|  | BD-SnPK2.5R | TCGACGGATCCCCGGGAATTCAACAGGTCCAATTTCTCCAC |
|  | BD-SnRK2.6F | ATGGCCATGGAGGCCGAATTCATGGATCGATCGGCGCTTAC |
|  | BD-SnRK2.6R | TCGACGGATCCCCGGGAATTCTTATAACGCGTATACAATC |
|  | BD-SnRK2.7F | ATGGCCATGGAGGCCGAATTCATGGATCGATCTGCGCTTAC |
|  | BD-SnRK2.7R | TCGACGGATCCCCGGGAATTCTCACATTGCATAAACTATC |
|  | BD-SnRK2.1FS1 | ATGGCCATGGAGGCCGAATTCATGGAGGAGAAGTACGAAC |
|  | BD-SnRK2.1RS1 | TCGACGGATCCCCGGGAATTCTTATCTATGGCAGATTTGCATGAA |
|  | BD-SnRK2.1FS2 | ATGGCCATGGAGGCCGAATTCATGGATTTGAAGCTTGAAAACACT |
|  | BD-SnRK2.1RS2 | TCGACGGATCCCCGGGAATTCTTATGGTGAAAATGTCGGGTTTTC |
|  | BD-SnRK2.1FS3 | ATGGCCATGGAGGCCGAATTCATGCAAAGTGTTGAAGATATTATG |
|  | BD-SnRK2.1RS3 | TCGACGGATCCCCGGGAATTCCATTTGAGCAGAATAATCAATC |
|  | BD-SnRK2.2FS1 | ATGGCCATGGAGGCCGAATTCATGGTCTTCCCTCCAAATC |
|  | BD-SnRK2.2RS1 | TCGACGGATCCCCGGGAATTCTTATCTATGGCAAATTTGCATTGA |
|  | BD-SnRK2.2FS2 | ATGGCCATGGAGGCCGAATTCATGGATCTCAAACTAGAAAATGTG |
|  | BD-SnRK2.2RS2 | TCGACGGATCCCCGGGAATTCTTAGGAAACATTGTTTTCCTTGTC |
|  | BD-SnRK2.2FS3 | ATGGCCATGGAGGCCGAATTCATGCAAAGCATCAAAGAAATAAAA |
|  | BD-SnRK2.2RS3 | TCGACGGATCCCCGGGAATTCTAATGCACAAACAAAATCC |
|  | BD-SnRK2.3FS1 | ATGGCCATGGAGGCCGAATTCATGGAAAGATATGAACTTG |
|  | BD-SnRK2.3RS1 | TCGACGGATCCCCGGGAATTCTTATCTGTGGCAAACTTCCATTGA |
|  | BD-SnRK2.3FS2 | ATGGCCATGGAGGCCGAATTCATGGATCTTAAGCTTGAAAACACA |
|  | BD-SnRK2.3RS2 | TCGACGGATCCCCGGGAATTCTTAATACGCATTGATGGTGTTAGA |
|  | BD-SnRK2.3FS3 | ATGGCCATGGAGGCCGAATTCATGCAAAGTGACGATGAAATACTG |
|  | BD-SnRK2.3RS3 | TCGACGGATCCCCGGGAATTCCAATGAGCAACCATAATCATC |
|  | BD-SnRK2.4FS1 | ATGGCCATGGAGGCCGAATTCATGGAAATTTGTCACAGAG |
|  | BD-SnRK2.4RS1 | TCGACGGATCCCCGGGAATTCTTAATCACGACTTGCATTCTCATA |
|  | BD-SnRK2.4FS2 | ATGGCCATGGAGGCCGAATTCATGCAATCACCTCAAGGTGTTGAT |
|  | BD-SnRK2.4RS2 | TCGACGGATCCCCGGGAATTCACCTTGGCCACCATAATCTC |
|  | BD-SnRK2.5FS1 | ATGGCCATGGAGGCCGAATTCATGGAGAAGTACGAGCTTG |
|  | BD-SnRK2.5RS1 | TCGACGGATCCCCGGGAATTCTTATCTCTATGACAAATACCCATT |
|  | BD-SnRK2.5FS2 | ATGGCCATGGAGGCCGAATTCATGTCTGAAACTAGAAAACAATCT |
|  | BD-SnRK2.5RS2 | TCGACGGATCCCCGGGAATTCTTACAGTGATTGGTCAAAGCTTGC |
|  | BD-SnRK2.5FS3 | ATGGCCATGGAGGCCGAATTCATGCAAAGTGTCGATGAGGTCAAC |
|  | BD-SnRK2.5RS3 | TCGACGGATCCCCGGGAATTCAACAGGTCCAATTTCTCCAC |
|  | BD-SnRK2.6FS1 | ATGGCCATGGAGGCCGAATTCATGGATCGATCGGCGCTTAC |
|  | BD-SnRK2.6RS1 | TCGACGGATCCCCGGGAATTCTTACCTATGGCATACTTGCATATT |
|  | BD-SnRK2.6FS2 | ATGGCCATGGAGGCCGAATTCATGGACTTGAAACTGGAAAACACA |
|  | BD-SnRK2.6RS2 | TCGACGGATCCCCGGGAATTCTTACGTGGGCTGATCGGGCCCACG |
|  | BD-SnRK2.6FS3 | ATGGCCATGGAGGCCGAATTCATGCAAAGTGTTGATGAAATCATG |
|  | BD-SnRK2.6RS3 | TCGACGGATCCCCGGGAATTCTTATAACGCGTATACAATC |
|  | BD-SnRK2.7FS1 | ATGGCCATGGAGGCCGAATTCATGGATCGATCTGCGCTTAC |
|  | BD-SnRK2.7RS1 | TCGACGGATCCCCGGGAATTCTTAACGATGGCATACTTGCATATT |
|  | BD-SnRK2.7FS2 | ATGGCCATGGAGGCCGAATTCATGGACTTGAAACTAGAGAACACA |
|  | BD-SnRK2.7RS2 | TCGACGGATCCCCGGGAATTCTTAAGTCGACTCATCCGCCCCTCC |
|  | BD-SnRK2.7FS3 | ATGGCCATGGAGGCCGAATTCATGCAGAGTGTTGATGAAATAATG |
|  | BD-SnRK2.7RS3 | TCGACGGATCCCCGGGAATTCTCACATTGCATAAACTATC |
| Y2H (AD ) | AD-bZIP1F | GCCATGGAGGCCAGTGAATTCATGAGTTCGTTTATCAACTTC |
|  | AD-bZIP1R | ATGCCCACCCGGGTGGAATTCCTACCATGGCCCTGTTAATG |
|  | AD-AREB1F | GCCATGGAGGCCAGTGAATTCATGAACTTCAAAAGCTTCGG |
|  | AD-AREB1R | ATGCCCACCCGGGTGGAATTCTCACCATGGCCCCGATTGTG |
| BiFC(pXY106-nYFP) | pXY106-SnRK2.1F | ATCCTCTAGAGTCGACATGGAGGAGAAGTACGAAC |
|  | pXY106-SnRK2.1R | TGCCTGCAGGTCGACCATTTGAGCAGAATAATCAATC |
|  | pXY106-SnRK2.2F | ATCCTCTAGAGTCGACATGGTCTTCCCTCCAAATC |
|  | pXY106-SnRK2.2R | TGCCTGCAGGTCGACTGCACAAACAAAATCC |
|  | pXY106-SnRK2.3F | ATCCTCTAGAGTCGACATGGAAAGATATGAACTTG |
|  | pXY106-SnRK2.3R | TGCCTGCAGGTCGACCAATGAGCAACCATAATCATC |
|  | pXY106-SnPK2.4F | ATCCTCTAGAGTCGACATGGAAATTTGTCACAGAG |
|  | pXY106-SnPK2.4R | TGCCTGCAGGTCGACACCTTGGCCACCATAATCTC |
|  | pXY106-SnPK2.5F | ATCCTCTAGAGTCGACATGGAGAAGTACGAGCTTG |
|  | pXY106-SnPK2.5R | TGCCTGCAGGTCGACAACAGGTCCAATTTCTCCAC |
|  | pXY106-SnRK2.6F | ATCCTCTAGAGTCGACATGGATCGATCGGCGCTTAC |
|  | pXY106-SnRK2.6R | TGCCTGCAGGTCGACTAACGCGTATACAATC |
|  | pXY106-SnRK2.7F | ATCCTCTAGAGTCGACATGGATCGATCTGCGCTTAC |
|  | pXY106-SnRK2.7R | TGCCTGCAGGTCGACCATTGCATAAACTATC |
| BiFC(pXY104-cYFP) | pXY104-bZIP1F | CGGTACCCGGGGATCCATGAGTTCGTTTATCAACTTC |
|  | pXY104-bZIP1R | CGACTCTAGAGGATCCCCATGGCCCTGTTAATG |
|  | pXY104-ABRE1F | CGGTACCCGGGGATCCATGAACTTCAAAAGCTTCGG |
|  | pXY104-ABRE1R | CGACTCTAGAGGATCCCCATGGCCCCGATTGTG |
| Vector primer | M13F | CAGGGTTTTCCCAGTCACG |
|  | M13R | GAGCGGATAACAATTTCACAC |
|  | G1F(35SF) | GACGCACAATCCCACTATCC |
|  | G1R | TGAACTTGTGGCCGTTTACGTC |
|  | T7F | GTAATACGACTCACTATAGGGCGA |
|  | 3BD | TTTTCGTTTTAAAACCTAAGAGTC |
|  | 3AD | AGATGGTGCACGATGCACAG |
|  | pB42ADF | CCAGCCTCTTGCTGAGTGGAGATG |
|  | pB42ADR | AAGCCGACAACCTTGATTGGAG |
|  | 0800R | CGACGGTATCGATAAGCTTGATATC |
|  | 104F | ATGACGCACAATCCCACTATC |
|  | 106R | TTTCCCAATGCCATAATACTC |

| **Table S2 Summary of transcriptomes from three tissues (flower, root, leaf) *T. mongolicum* samples.** | | | | | | | |
| --- | --- | --- | --- | --- | --- | --- | --- |
| **Sample** | **Raw reads** | **Raw bases** | **Clean reads** | **Clean bases** | **Error rate (%)** | **Q30 (%)** | **GC content (%)** |
| Flower_1 | 49891464 | 7533611064 | 49536062 | 7285074472 | 0.0239 | 95.25 | 45.02 |
| Flower_2 | 49245202 | 7436025502 | 48944364 | 7214268204 | 0.0239 | 95.23 | 45.04 |
| Flower_3 | 46804744 | 7067516344 | 46483018 | 6854567368 | 0.0239 | 95.25 | 44.23 |
| Leaf_1 | 48842144 | 7375163744 | 48572834 | 7183288284 | 0.0237 | 95.45 | 45.71 |
| Leaf_2 | 49374398 | 7455534098 | 48994470 | 7209031990 | 0.0241 | 95.02 | 45.42 |
| Leaf_3 | 52077866 | 7863757766 | 51510584 | 7576141002 | 0.0238 | 95.32 | 45.69 |
| Root_1 | 51391120 | 7760059120 | 51061994 | 7535400525 | 0.024 | 95.12 | 45.13 |
| Root_2 | 50869568 | 7681304768 | 50476942 | 7438482911 | 0.024 | 95.07 | 45.11 |
| Root_3 | 46366408 | 7001327608 | 46018906 | 6797016481 | 0.0243 | 94.83 | 45.03 |

| **Table S3 Summary of the sequence assembly results** | | |
| --- | --- | --- |
| **Type** | **Unigene** | **Transcript** |
| Total number | 77035 | 129,635 |
| Total base | 70791837 | 133,310,721 |
| Largest length (bp) | 20797 | 20797 |
| Smallest length (bp) | 201 | 201 |
| Average length (bp) | 918.96 | 1028.35 |
| N50 length (bp) | 1577 | 1601 |
| GC percent (%) | 40.29 | 40.06 |

| **Table S4 Statistics of annotations for assembled unigenes in six public databases.** | | | | |
| --- | --- | --- | --- | --- |
| **Data base** | **All_Unigene number** | **percent %** | **All_Transcript number** | **percent %** |
| GO | 33635 | 43.66 | 64290 | 49.59 |
| KEGG | 21091 | 27.38 | 36955 | 28.51 |
| COG | 40360 | 52.39 | 71567 | 55.21 |
| NR | 40732 | 52.87 | 77125 | 59.49 |
| Swiss-Prot | 33933 | 44.05 | 61093 | 47.13 |
| Pfam | 34035 | 44.18 | 60460 | 46.64 |
| Total_anno | 47295 | 61.39 | 84370 | 65.08 |
| Total | 77035 | 100 | 129635 | 100 |

**Table S5. Promoters and CDS sequences of TmSnRK2s in T. mongolicum**

TmSnRK2.1(CDS)

ATGGAGAAGTACGAGCTTGTAAAGGATATAGGATCTGGTAACTTCGGAGTTGCGAGGTTGATGAGGAACAAGGTTACTAAAGAGCTTGTTGCTATGAAGTATATAGAAAGAGGACACAAGATTGATGAGAATGTTGCTAGAGAAATCATAAATCATAGATCCCTTCGTCACCCAAACATAATTCGATTTAGAGAGGTAGTACTCACCCCCACCCACCTTGCTATTGTCATGGAGTATGCTGCAGGCGGGGAGCTGTTTGAGAGAATTGTCAATGCCGGAAGATTCAGTGAAGACGAGGCTAGATACTTCTTCCAACAGCTTATATCAGGAGTTCATTACTGCCATTTCATGCAAATCTGCCATAGAGATTTGAAGCTTGAAAACACTCTTTTAGATGGAAGCCCTGCACCACGTCTAAAAATCTGTGATTTTGGCTACTCAAAGTCATCACTGTTACACTCCAGACCCAAATCCACAGTGGGCACCCCTGCTTACATTGCTCCTGAAGTTCTTTCTCGTAGAGAATATGATGGCAAGACAGCAGATGTTTGGTCTTGTGGAGTTACACTTTACGTGATGCTAGTTGGAGCATACCCTTTTGAAGACCAAGAAGATCCCAAAAATTTCAGGAAAACAATTAATCGAATAATGGCTGTTCAGTACAAGATCCCAGACTATGTTCATATATCTCAAGACTGCAGACATCTTCTTTCAAGAATATTTGTTACCAATGCATCCAGGAGAATTACCCTAAATGAAATCAAAACCCATCCATGGTTCTTGAAGAACTTACCTCGTGAACTAACAGAAGCATCACAGTCTGTTTATTACCGAAAAGAAAACCCGACATTTTCACCACAAAGTGTTGAAGATATTATGAAGATTGTGGAAGAAGCAAGAACACCTCCGCCAGTTTCCAAATCACATGGAGCGTATGGATGGGGAGATGATGATGAAGATGATGATGATGATGATGGTAAAGAAGCACATGATGGTGATGATGATGATGGGGATGAAGATGAGTATGATAAGAGAGTGAAAGAAGCTCATGAAAGTGGAGAAATTGGACCTGTTTAA

TmSnRK2.1(Promoter sequence)

ACACAAAACCATTTACCAAAACATCAAACATCTAAAAATATAATAATAAATCTTTGCCCAAATTTGAAATCACACACCAATGACCAAAACACACCTATTTGACAATACCAATTTACAGACTCACTTTCCATTATTCAATCCCCATTTTTTTTACACAAATCCTCTCTTCCAACATCAAATTATCAAACAGGTCCAATTTCTCCACTTTCATGAGCTTCTTTCACTCTCTTATCATACTCATCTTCATCCCCATCATCATCACCATCATGTGCTTCTTTACCATCATCATCATCATCATCATCTTCATCATCATCTCCCCATCCATACGCTCCATGTGATTTGGAAACTGGCGGAGGTGTTCTTGCTTCTTCCACAATCTTCATAATATCTTCAACACTTTGTGGTGAAAATGTCGGGTTTTCTTTTCGGTAATAAACAGACTGTGATGCTTCTGTTAGTTCACGAGGTAAGTTCTTCAAGAACCATGGATGGGCTTTGATTTCATTTAGGGTAATTCTCTGTAGAAAAATTACAATTATTTTTTCATTATAAATAACTAAATATAAACATGTCAATATACCCTGGATGCATTGGTAACGAATATTCTAGAAAGAAGATGTCTGCAATCTTGAGATATATGAACATAGTCTGGGATCTTGTACTGAACAGCCATTATTCGCTGAGTAAATAAAAGTCAAAGGTCAAAGGTCAAAGTTTGTCAGAAAGAAAATAAAGTAAAAAAAAGACTGACATTAATAGTTTTCCTGAAATTTTTGGGATCTTCTTGGTCTTCAAAAGGGTATGCTCCTACTAGCATCACATAAAGTGTCACTCCACAAGACCAAACATCTGCTGTCTGAATACCATAAAACCAAAACATGGTATTTTGAAGTTTAATTGTGAATTATAAATAAAAATAAAAACATGTGAAGTATTGATGATTATGATTTATGAGAGTGAAGATGTTTTGGGGTTTTAGTACCTTGCCATCATATTCTCTACGAGAAAGAACCTCAGGAGCAATGTAAGCAGGGGTGCCAACTGTGGATTTTGGTCTGGAGTGTAACAGTGATGACTGAATAACAACTGAAGCCATTAGGATCTTGGTTTTGATTACAGAATAAGTTGACAATGGATGGATGGTGATTACCTTTGAGTAGCCAAAATCACAGATTTTTAGACGTGGTGCAGGGCTTCCATCTAAAAGAGTGTTTTCAAGCTTCAAATCTCTATGGCAGATTTGCTGTAGAGCATTTTATGAAGTTAAACTCATATGTAAAAAGTGAAAGTGCAATTGTGGAAATACGATTGTGATTTTACCATGAAATGGCAGTAGTGAACTCCTGATATAAGCTGTTGGAAGAAGTATCTAGCCTGGATGATTGACAGATAATATTAATACAGTGAGGATTGAAGAATAAGGAAGCAATCTCCATGAAATAAAACTGTTTTCAGTTGTTATGTTGTTATGACCTCGTCTTCACTGAATCTTCCGGCATTGACAATTCTCTCAAACAGCTCCCCGCCTGCAGCATACTCCATGACAATAGCAAGGTGGGTGGGGGTGAGTACTACCTATTGAACACCACAGAGATGATGTAAAATTGCAATCCATGGTGCTATTGAAAGGATGTCGGCTTGATGAGAAGAAGAAAACAATTGGACAAGTTTACCTCTCTAAATCGAATTATGTTTGGGTGACGAAGGGATCTATGATTTATGATTTCTCTAGCAACATTCTCATCAATCTACAACCAAAACAGGACAAAAACCCTTGTAGTTTAGCATAAGAAGAATCTTTGAAAACAATATGAATTGGAATAAGATAACATTAAAATTGAGTATAATTGAGAGTAGAGATATGGAGAAAACCGTATATACCTTGTGTCCTCTTTCTATATACTTCATAGCAACAAGCTCTTTAGTAACCTTGTTCCTCATCAACCTCGCAACTCCGAAGTTACCAGATCCTATATCCTTTACAAGCTCGTACTTCTCCAT

TmSnRK2.2(CDS)

ATGGTCTTCCCTCCAAATCATGTCGACCACCCGACAAAGGCATTTATCGTTGTAGCATACGTACAAACACGCAAGCTTCACCGTGCACTCACCAAAACTTGGCATCAAAGAGACTTCCTCAATCGTTTTTATGATTATTTGTTGAATGTAATGGAAAGGTACGAGGAAATAAAACACATAGGATCAGGGAACTATGGCGTAGCAAAACTTGTGAAGGATAAACAATCTGGCGAGCTTTATGCAGTCAAGTATATTGAGCGAGGACCTAAGATTGATGAACATGTAGAAAGGGAGATTTTGAACCACAGATCACTTAAGCACCCCCATATTATCCGATTCAAGGAGGTGTTACTTACAGAAACTCATCTTGCCATAGTGATGGAGTATGCAGCTGGAGGAGAACTGTTCGATAGAATTTTTAATGCTGGCAGATTCAGTGAAGACGAGGCAAGATTTTTCTTCCAACAGCTAATATCAGGCGTCAGTTACTGCCATTCAATGCAAATTTGCCATAGAGATCTCAAACTAGAAAATGTGCTTTTGGATGGGAGTTCAACACCACGTCTTAAAATTTGCGACTTCGGATACTCTAAGTCCTCGGTGTTCCACTCGCAACCCAAATCTACGGTTGGGACGCCTGCATATATTGCACCCGAAGTCCTATCTAGAAAAGAATACGATGGCAAGATGGCAGATGTTTGGTCTTGTGGGGTTACGTTGTATGTGATGCTTGTGGGCGCTTATCCTTTCGAAGATGAAGATGATCCGAGAAACTTCCGAAAAACACTTACCCGTATACTTAGTGTCCACTACTCAGTCCCAGACTACGTTCGAGTTTCTGTTGGCTGTAAACACCTTCTGTCTCGGATATTTGTAGCCAACCCCGATAAGAGAATAACCATACCAGAGATCCAGAAGCATCCGTGGTTCTTGAAGGAGTTGCCAAGTGAACTCATTGATGAAGATGAATCCATCTCCGATAATGACAAGGAAAACAATGTTTCCCAAAGCATCAAAGAAATAAAAGAAATACTACAAGAGGCAAGAAGATGTACACTGCGGACCAAAGATGAAGGGGGGAGAATGGATCTTGAAGATGATATTGATACGGATGCTGAGATCGAGGATGATGCAGAGACAAGTGGGGATTTTGTTTGTGCATTATAA

TmSnRK2.2(Promoter sequence)

GGTTTTCTTTATTACTTGTCCAATTCCAATAATTCTAAGTGCAATAAGCTTATATATGATGAAAAATGAGTTGTACAAAAGTTGAAAATATTTGGAAACTCCCAAGGATATTTTGGTCATTTTGATGACCTTTAGGATATTGTTTTTGTAGAATACAAGTGAAGGGAGTTTCTAGAAGGAGGTATAAGAAGAGTACAAGCTATCTAGGGACTCCTGCAAGCTAAAGCGAGTACGTGTGATTGGTTTTCCCTCTTTTGAGGATACATGGCAAAACCTATTTTGAAAAAGTATGTTGCCTTTGTGAAGATAAATTTTTTATGCTTCTATGATGATATGTAGAATGTAATGGAGATGTGAGAACTAGAAAGAATATATCAGAAGTGCACGAGTACATTGTCCGGACCATGAGAGTCTCGCAAGGGGGCCTAGAGAATTCTATTGGGATGGTACCTCCCTTCACGAAATAGAACTCCTAATCTATATGAGAGTCTCGGTGAACTGATTTCGTTCTTTTCACCTTGGCCTAGACTTGGGGTGGTTTTTTTTTTTAACATAGACCCTAAGTATAAAATAATAGCAACGGTTGATCACCGGAATTAGTCATTGATATCTTACGGGTGATGTCACATGATTTAGCTAGAATTGATGATTATGTTATTGTTGATTATGATAAATTAATTTCATATGAATATTTTGGGATTAAAACCCGCGTATCTCACCAGACTTTATCTGACGTACTGTTTTTGTGTCATGTGTCTCAGGGGATAGTTCTCATGCTTAGGAGAATTGGACTACTTGAAGTGTTGTTAGCAAGTGGAATGAAGACTTGGTTGTTATCCATTCTCCAATTAGGTTACACTATGGTTGTTTTGATACATTTTGTTAAATACCCCAAACTTTAATTATGTTTTTATGATGTCTTTTGTTAAACCATGATATCAAAAATGTATGAAAAAGTTTGAAATTTTTGGGGTGTTTCAGCAGTGACCAAAATCGCAAAAAATATCCAATATTGGACCCGTTGTACACCAGCAAAACTTTTTGGACCAAAAATGCATAATTTTGACAACCACAGGACCATTTTTGTAATTTTTTCAAATTAAAATAAACTTGGAAACATAATAATAAAATGAAATATATCATTATTTTAATGTTCATAAATTCAGGAGTGCATCCATTTATATAGGGTCTGTCTGGATCTTCAAGGGTATCGTTTGTTGATACTTGATATAATCAATTTTGAAGGATTCTTATTGTTTTACTTTCGTTCTGTATCAATATATATTGGGTGATGTGGGATCACCGGAGGAGGTCATCGGCGCTAATACGTAGAAGCCGTGTGGGGATAAAATGATAATTGATAAGGCGTGTAGGGTAAGGGTTAGGTGGTTGGTGGGACCATACTGTCTTAATTATAACATAAATAAGTATTAAAAAAAATATAGAGAACTATGATTCTAAGCATACGTTTATTTTGTTCTTAAATTACAAATTTAAAATAAAACACGTTTATTTTAATCTTAACGTACTTAATTCTCTGAGACCAAATCTAAAATAAATTCAAAGCATATAATTATAGAAAATGCGTAATTGAAAAGTACATATGGTCGAAGTTTGCAATTTTAACCCCAAACATGTTAATGGATAACTTATCTTATTCACTCATATAAAACTATAGAACAATTATACACATTTAAGTACGTTACAACCTAACATTTTGTAAATTATAAATCAAAAATATATATTATAAAAAAAGATATGTTTGTATGATGTTTACGTAATACATTCTACGAAACTTATCTAAGCTAGATTTTGTAAGTTCGAACTCAAGGTGCTTTTCTTTAAGTAGTCGACATCAATATATATCATGTTTTACTAGGAAACCAAGTGTTATCGACATCGTACACATCACAGCAAATAAAATAAATAAAATAATAATAATAATCTGTTACATCGGAAAACTAAATATAGTCCAGCCACTATTATCTTGCCACACGTAG

TmSnRK2.3(CDS)

ATGGAAAGATATGAACTTGTTAAAGAAATCGGGTCGGGTAGTTTTGGGGTAGCGAAGCTTGTTAGGGACAAGGGGACCAGAGAGCTATTTGCAGTCAAGTTTATCGAAAGGGGCAAAAAGATTGATGAACATGTACAGAGGGAAATTATGAATCATCGATCTTTGAGGCATCCGAATATTATCAGATTTAAAGAGGTGTTGCTAACACAAACTCATCTAGCAATAGTCATGGAGTATGCTGCCGGAGGAGAGCTTTTTGAGAGGATATGCAATGCGGGTAAATTTAGTGAAAATGAGGCAAGATTTTTCTTTCAACAACTTATATCAGGAGTTAGCTACTGTCATTCAATGGAAGTTTGCCACAGAGATCTTAAGCTTGAAAACACATTGCTTGATGGAAGTGCAGCTCCTCGTGTTAAAATATGTGATTTTGGGTACTCAAAGTCATCGGTTCTCCATTCACAACCAAAGTCAGCAGTCGGGACACCCGCTTACATTGCACCCGAAGTGCTCTCAAGAAAAGAATATAATGGAAAGCTTGCAGATGTTTGGTCATGTGGGGTTACTCTATACGTGATGCTAGTCGGGGAATACCCTTTTGGAGATCCGGATGATCCAAGAAACTTCCGTACAACTGTTTGCCGGATACTTAGTGTTCAATACGCAATCCCAGATACTGTAGAAATTTCAATGGAATGCAAACATCTTCTCTATCGGATCTTCGTTGCAAACCCTGAGAAGAGAATAACGATTCCAGAGATCCAAATGCACCCGTGGTTCTTGAAGAACTTGCCGACGGATTTAATGGCGGGAGGAAGCTCTAACACCATCAATGCGTATCAAAGTGACGATGAAATACTGTCGATTATACAGGAGGCGAGAACTCATCCTGGGATGCTTTGTGATGGATCGCCGCAGCTTCTTGGTGACAGTATGGATTTTGATGATTTGGATGATTCGGATATTGAAGATATAGATATAAGCGATGATTATGGTTGCTCATTGTAG

TmSnRK2.3(Promoter sequence)

ACTAAAATGCATCCATTGTATGTATAAATTGAAATAAAAGTTTAAGTATAAGTAAAAGTTTAGAAGTTATTCAATTTTTTTGTTGAATTCCAATTTATTTTTTGCGAACCAAATGCATGATAGGTTTCAATTCTTTTGACTTATTTCATTTCTGGCTAACACATAATGCGAACCAAATCACAATTAATTATATTTTAAGATAAATGAGAGGGGCTCACAAAAACAAAAAAGAAATAAAATTATTACCTTTGAGTACCCAAAATCACATATTTTAACACGAGGAGCTGCACTTCCATCAAGCAATGTGTTTTCAAGCTTAAGATCTCTGTGGCAAACTTCCTGCAATCAATGCATCAATTGGTCAAGAACCAAAAAGAGGTGGTAATATTGTGTATCAAAAGAAAGGCCATAAATGGAGTTTTTATATATACCATTGAATGACAGTAGCTAACTCCTGATATAAGTTGTTGAAAGAAAAATCTTGCCTGGTACACAAGAGGAAAAAAACGGCATAATCAAAACCATAAATCATCATGATATTTGTTTTTTAGTGTAAATGAGTTATTTTAAAGGGTTAATCACTATAATAACCAATACGGGGGAGATTAATATCACAAAATGACCACCTTTTTAAACTTCTATCACAAATTAGTCACTTATTTCGCGAATGGATTCCTCGAAATGTGTCTTTTAGTTCTTTTTCACAAAGAATTATTTTTTTGTGACAATGGTGTCATTTTGCAAATATAATGATGCCATTTTCAAGAAATTCTTAAGTTTTTTGTAGTGTGACCACTAACAAAACAATTTAATCTTGCGAAATACATGATCATTTTCTGATATAAGTCGAAAATGTGGTTATTTTATACATGATCATGTTACTTATCATTTTTGGTTCTTTTTCTAAACCTTAGATATGATAATTTTTGAATTAGCTAAACGTTATAAACTTATAACTATGTTAAGTTAAAACTTGCTAAAGTAAGCATATATTACAGTAAGAAGCCTAATAACATCTGAAGCAATTCAAGAATTCGACGGTTTGTTAAAGGAACTTGCCTCATTTTCACTAAATTTACCCGCATTGCATATCCTCTCAAAAAGCTCTCCTCCGGCAGCATACTCCATGACTATTGCTAGATGAGTTTGTGTAAGCAAAACCTGAAGAATATAGTAATATTACTTTCATATGCTTGTGTATTATAGCATCCTTTTTCTACTTTTTCTCTTACAACATTGTACTTTTATTAAAAAAAATCTTGTTAAGATATTGTATCTTGAAAACTATACTCAATAATAGCATTGGATTTTCAAAAAAAATCTTTCAAGGATATAATTCTTTGAAATTGGAAATTGCAAATTTTGGATACAATTCTTCTATGTTATTCTTGAAATTGGAAATTGCTTGGTTTGAAATTTGAATCTTTGATCAGAAGTTCACTTTTGGACTAAAGGAAGAACAGCAATAAAATTACTAGACGCGATCATGAAGAATTCAATAACAGAAACATTTTGGGGATGAACAATATACCAAATCCATACGAACAATTGAACAATTGAAGTCAAAGTTTGACTTTTTCAGATTAAGATAGAATAACAATCCAATTAAGCAACTTCAGTGAAGCATAATGAAACAAACATCGAAAAAACATCTCCGACACTAAAAACACACACACAATTAAAAGTTTAGAGCTAACCTCTTTAAATCTGATAATATTCGGATGCCTCAAAGATCGATGATTCATAATTTCCCTCTGTACATGTTCATCAATCTGCAATTTTTCAAAAATCGTAATCAAACCCAGAAAGCGTTTACCATCAAATCATTACATCAAGTGCATAAAAACATCAAAAGATTCAAAAAAGATCAAAAGAGACAACCTTTTTGCCCCTTTCGATAAACTTGACAGCAAATAGCTCTCTGGTCCCCTTGTCCCTAACAAGCTTCGCTACCCCAAAATTACCCGACCCGATTTCTTTAACAAGTTCATATCTTTCCAT

TmSnRK2.4(CDS)

ATGGAAATTTGTCACAGAGACCTTAAACTTGAAAACACTCTACTAGATGGAAGTCGATCCCCACGTCTCAAGATTTGTGATTTTGGCTATTCAAAGTCTGGCTTATTGCATTCGCAACCAAAGTCGACTGTGGGAACACCAGCTTATATTGCCCCTGAGGTTCTATCTCGTAAAGAATATGATGGCAAGATTGCAGATGTTTGGTCATGTGGGGTGACTCTTTATGTTATGCTTGTAGGAGCATACCCTTTTGAGGATCCAAGAGACCCTCGAAACTTCCGTAAAACAATTGGGCGAATCATGAGCGTTCAGTACTCGATACCAGATTATGTGCGTGTTTCAAAAGATTGTAGGCATCTTCTTTCTCATATATTTGTTGCTAACCCATCCAAGCGGATCACAATAGGAGAAATCAAGAAACATCCATGGTTTATGAAGAACATGCCAAAGGAGCTCGTTGAAGGTGAGAAGACAAATTATGAGAATGCAAGTCGTGATCAATCACCTCAAGGTGTTGATGAGCTCAATCGGATTATACAAGAGGCTATGAATCCTGGAGAAGGGTCTACCAGAAATGGAGAGATTGTCATAGGTGATGGATCATTGGATCCTGAAGATGAGATTGATTTGGATGATGAGATTGAATCTAGTGGAGATTATGGTGGCCAAGGTTGA

TmSnRK2.4(Promoter sequence)

AAATCATACATTTTGGTCAAACTTAAGGCTTTTTAATCATTACCCTATTTTCTATCCATTAAGCTTTGCAAACCAAACTTTATATATTATCACAAATTTCAAATATAAAACTAACATGTCCATTACAAATTATTACGAATGAATGTCTTACCCAAAATTTAATCTACACACACTTCGTATCTCATAAAACACAGTCATCCACGAAAAGAAAATTTCACACAAAAAAAATCGTAAAACACAATTTTGGAAATACGTATCATTTTAATAGAAATCGTTAACCTTGGCCACCATAATCTCCACTAGATTCAATCTCATCATCCAAATCAATCTCATCTTCAGGATCCAATGATCCATCACCTATGACAATCTCTCCATTTCTGGTAGACCCTTCTCCAGGATTCATAGCCTCTTGTATAATCCGATTGATCTCATCAACACTTTGAGGTGATTGATCACGACTTGCATTCTCATAATTTGTCTTCTCACCTTCAACGAGCTCCTTTGGCATGTTCTTCATAAACCATGGATGTTTCTTGATTTCTCCTATTGTGATCCGCTACAAGGACAGTAAAAAAAAGCTAATTTTCAGATAAATTGAACAATTAAGTTTGAGTTACATTGATGGAACAAACCTAAAATTTGAAAATTACTTAAAGGCATTAATCCTACATGTAATTACTTGTTTTAGATGTTTTATTAAAAATAAATAAGTAGCAGTACCTTGGATGGGTTAGCAACAAATATATGAGAAAGAAGATGCCTACAATCTTTTGAAACACGCACATAATCTGGTATCGAGTACTGAACGCTCATGATTCGCTACATATAACAAGAACAAATCAATGTTATAATCCAGTCAAAACATTTCAAGTTAGGTAAATTACACAAAAGACCCCAAGTTTATATGAAAATTCTATTTTGACATTGTGTAATTTTTTGTCTCAAATTTGGCACTCTATTATCAATTTGTCTTCAGATGTGACATTGACCCGACTAATAAGGGTTATTCTGGTAAAATTGAGTTTTCATGTCTTCATTCCGTCGTTCATATTAACCGATTGTATGATCACATTGTGTTTTTACACCTCTGTAATAGTTAATCTTATAAAGTGATTGTTTTTATCAAAACCGTAAATTTACGGGATTGATGTGTAAAAACAAAAATGTGACCATATAACCGGTTAATATGTGCGATGACATGGAGACATGAAAATTCAATTTTACCGGAATTGCCCTTATTAGTCGGGTTGATGTTACATTCGAAGACAATTTGATAATAGAGTGTCAAGTTTGAGACAAATAATAACACGGTGTCAAAATAGAAATTTTTATGTAAACTTGAAGTCTTTTGTGTAATTTACCCTTCAAGTTATAATGAAACTTCGATGCCTGTAGTTACTTTGAAATCAATTAAAGTTTTTAAGAAGTTTTTGGTCGTCATAAGGTTGTCCAATCCATTACTATCGTAGTCTTGAAATTACTAATTTTGACCGAAAGTTAACACATATATTTGTTCTTCCAGGAAGAAATAAATCTTACCCCAATTGTTTTACGGAAGTTTCGAGGGTCTCTTGGATCCTCAAAAGGGTATGCTCCTACAAGCATAACATAAAGAGTCACCCCACATGACCAAACATCTGCAATCTAACCACAAAACAAAAATAAAACAAAACTATGGTAATACTTTTACAATATTGTAACCAAAATTTAACAAATTACAATATAGTACCTTGCCATCGTATTCTTTACGAGATAGAACCTCAGGGGCAATATAAGCTGGTGTTCCCACAGTCGACTTTGGTTGCGAATGCAATAAGCCGGACTAGTCAAATGACAAAAAAGGACAATCAAGAATCATAAAATTTGCATAAAAATGTATTAAAAGATAAAAAAAATAGTATTTTAACCTTTGAATAGCCAAAATCACAAATCTTGAGACGTGGGGATCGACTTCCATCTAGTAGAGTGTTTTCAAGTTTAAGGTCTCTGTGACAAATTTCC

TmSnRK2.5(CDS)

ATGGAGGAGAAGTACGAACCTGTGAAAGATGGTTTTGGGAATTTTGAAGTGGCAAGGCTGGTTAGAGATAAGAAGACTAAAGAGGTGTTTGCCGTCATATACATTGACAGGGGGGAAAAGATTGATGAAAAGGTACACAAGGAAATCATTAACCATAAATTGTTAAGACATCCAAACATCGTGCGGTTCAAGGAGGTGTTGCTAACTCAAAAACGTTTGGCCATAGTCATGGAATATGCACATGGCGGTGAACTTTTTCGTAATATTAGGTCCCGTGGCAGATTCAGTGAAGATGAGGCTCGAGTTCTCTTCCAACAACTTATATCCGGAGTCAACTACTGTCATTCAATGGGTATTTGTCATAGAGATCTGAAACTAGAAAACAATCTCCTCGACGGGAGTCCATCCCCACGTCTCAAAATATTCGATTTCGGTCACTCAAAGTTTGGTTTATTGGAACCACAAGCGAAATCTAGGGGAACGCCAGCATACATTGCCCCTGAGGTTCTATCTCGGAAAGAATATGATGGGAAGATCACAGATGTTTGGGCATGTGGGGTGACTTTGTATGTTATGTTGGTTGGAGCTTACCCTTTTGAGGACCCCGAAGATTCTAGAAATTTTCGTAAAAGCATGGAGAGAATCATGAACGTACAATACTCGATACCAGATGATGTGTGTGTTTCTGTTGATTGTAGACACCTTCTTTCTCATATTTTACTTGCCAACCCCGACAAGAGGATCACAATAGCAGAAATCAAGAAACATCCATGGTTTATGAAGAACATGCCTAAGGATCTAGTTGAAGGTGAGAAAACAAACTATGAGAATGCAAGCTTTGACCAATCACTGCAAAGTGTCGATGAGGTCAACCATATCATACAAATGGCAAAAGTTCCCGGTGAAGGCTCCACCACCAGGGATGGGAGGGAGGAGATTGGTGGGTCCATGGATCCTGATGATGATGATGATGATGATGACTTTGACTTGCAGAATGAGATTGATTATTCTGCTCAAATGTGA

TmSnRK2.5(Promoter sequence)

TTAAACATCAATTTTAAGTTCAGTTATTTGATTTTTTTTGGGGAAAAACATCATAATGTTTTTGGAGTCGGAACAGAAAACAAAGACAAGATGATACGTCTTGAATATGTGCACAAATCTCACCTCCATGCTTTTACGAAAATTTCTAGAATCTTCGGGGTCCTCAAAAGGGTAAGCTCCAACCAACATAACATACAAAGTCACCCCACATGCCCAAACATCTGTGATCTAGCACAAAGAAATTCACAATTTTATTATAAAAAAGGGCTATGGTCGAAGGTCACTAAGGTTGTTGTCGCGGGTCAGAAGGACCATACTGGCTTAACAAAATTTAGTGACTAAATAAGCAAAACTACAAATTTAATGCCTAATGTTGGCCTTAACTTTAAAAAAACAAAAACTTTAGTTAACCGTTAATTTAACTATACAAAACGAAACAATACCTTCCCATCATATTCTTTCCGAGATAGAACCTCAGGGGCAATGTATGCTGGCGTTCCCCTAGATTTCGCTTGTGGTTCCAATAAACCAGACTGCTCAAAACAACCATAAAAATATAGAAACAAAAAAGGGAAAACATACAAAAATGTTAACTTTGGTTTAGAAGTTGACAATTTGAGAATACTAAACCTTTGAGTGACCGAAATCGAATATTTTGAGACGTGGGGATGGACTCCCGTCGAGGAGATTGTTTTCTAGTTTCAGATCTCTATGACAAATACCCTGAATTAAAGAAACTATAAGGTAATATATCATTTTTACCATAAAAGGAAAGACATTTTCTTTTACCATTGAATGACAGTAGTTGACTCCGGATATAAGTTGTTGGAAGAGAAATCGAGCCTGTATTCAAGAAAATAAATGTCCTAGCTGTAATGCTTTTGAAAAATTAATAAAAAGACAAAGACAGATGAAGTTAAAAAACGTGTGCAAAACCTCATCTTCACTGAATCTGCCACGGGACCTAATATTACGAAAAAGTTCACCGCCATGTGCATATTCCATGACTATGGCCAAACGTTTTTGAGTTAGCAACACCTGCAAGATTTGCATTTAGAAATATGAAGAATGCCATGTTTCTTTCATGAAGAAGACATTCACGTCTTTTGCACAAACGAGTGATCAATAGCGAGTATATGTCCTATATTGTTCTATCTTTTTAGTGGGACAAAGACATCAGTCACAGTCACATTAAACACACTAAGCTAAAAGCTGAGATTTACCTCCTTGAACCGCACGATGTTTGGATGTCTTAACAATTTATGGTTAATGATTTCCTTGTGTACCTTTTCATCAATCTTACATAATTAAAACAACAAAGTTAGTTGTCATAAGTTGCTATTGAAGTGGAAATGCATGAGACGAGTATATGTGATCTTTATGAGAAATATTTACACTAATTAACAATTACACGATGTCTTTTACTTAACAAAATTTTAAAATGGGTTTTCAAATCTTTAAACATGTCTTAACTTTAGCTCAAAACTATGGGTGCTCACAAGCATACAATAAACCTCATTATAACAAATTTGATTGGTTGGTTCATGGTTGTATCCTATCAAGTGTGATAACTTGTTCAATACCTTTATTTTCTGTTTAGAATGTCAAATACACACGCAACCATTATCGTTAGACTATTGGCTGATGGACACATTTCTGTCAACTTCTGATATTGCTAGACTATAACCCTCAACTTTTAGTTGATGAACACATTTCTGTCAACTCGGATTCGGGTTATAATGGTCGAATATGTTTCCACATTTTCGGATTCCATTGTTCGGGTCCATGTTTAACTTAACCAGACACATGTAATATATTTTCCAAACATTAATTCCAACAAAATAGTAAAACGTAGAAACTCAAATGGAAAGTAACTTATACCTTTTCCCCTCTGTCAATGTATATGACGGCAAACACCTCTTTAGTCTTCTTATCTCTAACCAGCCTTGCCACTTCAAAATTCCCAAAACCATCTTTCACAGGTTCGTACTTCTCCTCCAT

TmSnRK2.6(CDS)

ATGGATCGATCGGCGCTTACGATGGGTCCGGGTATGGATATGCCGATTATGCATGATAGCGATCGGTACGAGCTTGTGAAAGACATTGGTTCAGGTAACTTCGGCGTCGCGAGGCTGATGAGGGATAAACTGAGTAACGAGCTTGTTGCAGTTAAGTACATCGAGAGAGGTGAGAAGATTGATGAAAATGTGCAAAGGGAAATCATTAACCACAGATCACTAAGGCACCCAAACATCGTTAGATTCAAAGAGGTTATTTTGACACCAACACATCTGGCTATTGTTATGGAGTATGCATCTGGTGGAGAACTTTTTGAGAGAATATGTAACGCAGGGAGGTTTCCAGAAGATGAGGCACGTTTCTTTTTCCAGCAACTTGTATCTGGAGTGGGTTATTGTCATAATATGCAAGTATGCCATAGGGACTTGAAACTGGAAAACACACTTCTAGATGGTAGCCAAGCTCCAAGACTCAAGATTTGTGATTTTGGATATTCCAAGTCGTCTGTTCTCCATTCACAACCAAAATCAACGGTTGGAACACCCGCATATATTGCCCCCGAAGTTTTACTAAAAAAAGAATATGATGGCAAGATTGCAGACGTTTGGTCATGTGGTGTGACATTATACGTCATGCTTGTGGGAGCTTACCCTTTTGAAGACCCCGAAGAGCCTAAGAATTTTCGCAACACAATACAGCGAATTCTTAACGTTCAGTACTCCATTCCAGCCTACGTTCATATATCCACCCCATGCCGCAATTTGATCTCCAGAATATTTGTCGCTGAACCTGCTAAGCGGATAACAATGGACGAGATTAGAAACCACGAATGGTTTATCCGGAACCTTCCGGGAGACCTAATGAACGAAAACGCAATGGACCAATTTCGTGGGCCCGATCAGCCCACGCAAAGTGTTGATGAAATCATGCAGATAATAGCGGAAGCTACAATTCCACCAGCCGGGGCCCACAACCTCAACCAGTATCTGACTGGCAGCCTGGACATCGATGATGACATGGACGAGGATCTTGAAAGTGATCCGGATCTTGATATTGACAGCAGTGGGGAGATTGTATACGCGTTATAATGA

TmSnRK2.6(Promoter sequence)

ATCTCCCCACTGCTGTCAATATCAAGATCCGGATCGCTTTCAAGATCCTCGTCCATGTCATCATCGATGTCCAGGCTGCCAGTCAGATACTGGTTGAGGTTGTGGGCCCCGGCTGGTGGAATTGTAGCTTCCGCTATTATCTGCATGATTTCATCAACACTTTGCGTGGGCTGATCAGGCCCACGAAATTGGTCCATTGAGTTCTCGTTCATTAGGTCTCCCGGAAGGTTCCGGATAAACCATTCATGGTTTCTAATCTCGTCCATCGTTATCCGCTATTTAAAAAAAATCATGTCAGTAATAAATAACAGAAAATTCGGGCCATGTAGGCCCCAAACACTTCGATAATGAGACAAAAACTGCACTTTTTTGTCTTACACAAAAAGTGACTTAAAGTGCACTTTTTTTGTCTTACACAAAAAGGTGTTAGATACCCTGATGTCAATAGGACAAAAATTGCACATTTTTGTTGCATACAAAAATGTGACAACTGTGATTGTGATTGTGACTGTGAATCAGACTGTCACCTTAGCAGGTTCAGCGACAAAAATTCTGGAGATCAAATTGCGGCATGGGGTGGATATATGAACGTATGCTGGGATTGAGTACTGAACGTTAAGAATTCGCTGAAAAAAATAAACTATCACTTGAGTCAAGTATTGCAAATAGGGATGAAAGTATGTTTGCAAAAAAATACGATTGTGTGTAGTATTTTATAATTTATACCTGTATTGTGTTGCGAAAATTCTTAGGCTCTTCGGGGTCTTCAAAAGGGTAAGCTCCCACGAGCATGACGTATAATGTCACACCACATGACCAAACGTCTGCAATCTAAATTCATTGTTTTATATTATAAAAAGGACAGGACATGACATGACATGACAGACTGTGTTTGACTGTGATGCATAGAGAAGATGATGTTGCTAACCTTGCCATCATATTCTTTTTTTAGTAAAACTTCAGGGGCAATATATGCGGGTGTTCCAACCGTTGATTTTGGTTGTGAATGGAGAACAGACGACTGATTAAATCAAGATTCAAGAATCGCGTAATATTAATTTCCAAAACATTAATGAAAAAAAATAAAAAAAATAAAAAAGATGTGAGATTTTGTACCTTGGAATATCCAAAATCACAAATCTTGAGTCTTGGAGCTTGGCTACCATCTAGAAGTGTGTTTTCCAGTTTCAAGTCCCGATGGCATACTTGCTACATTAAACACAAAATCATATATTACATAATCTTTGTGTTATCACTTTATAAATTACATAAAAACAATACTAATTCAATTATTCAATTATTCAATACCATATTATGACAATAACCCACTCCAGATACAAGTTGCTGGAAAAAGAAACGTGCCTGCATTACATTTACAATTTACAATTAACATTTAACGCCAATATTTTAAATTTTATAACAACAAACATAAATAACAACAAATCAATGCATTAAAAATACAAACCTCATCTTCTGGAAACCTCCCTGCGTTACATATTCTCTCAAAAAGTTCTCCACCAGATGCATACTCCATAACAATAGCCAGATGTGTTGGTGTCAAAATAACCTGGAAACCAGATAGAAATTTATTACTAAAAGAAGGCGTTTTTAGCTTTCTGAAAGAGAAATTGGTTTTGAAGTTTGAAGAAAGAAACCTCTTTGAATCTAACGATGTTTGGGTGCCTTAGTGATCTGTGGTTAATGATTTCCCTTTGCACATTTTCATCAATCTGTTGGGTAACAGTAAGTGAATGATCATTGATAGATATGTAATTATGCAAGAAGCAAATTCGAAATCAAAATTCTTCAAATTTGAACACTGACCTTCTCACCTCTCTCGATGTACTTAACTGCAACAAGCTCGTTACTCAGTTTATCCCTCATCAGCCTCGCGACGCCGAAGTTACCTGAACCAATGTCTTTCACAAGCTCGTACCGATCGCTATCATGCATAATCGGCATATCCATACCCGGACCCATCGTAAGCGCCGATCGATCCAT

TmSnRK2.7(CDS)

ATGGATCGATCTGCGCTTACTGTGGGTCCGGGTATGGATATGCCTATCATGCACGATAGTGATCGCTACGAGCTAGTGAGAGACATTGGTTCTGGTAATTTTGGTGTGGCGAGGTTGATGAGGGATAAACAGACTAACGAGCTTGTTGCAGTCAAGTACATCGAGAGAGGTGAGAAGATTGACGAAAATGTACAAAGGGAAATCATTAACCACAGATCACTAAGGCATCCAAATATCGTCAGATTCAAAGAGGTCATCTTAACACCAACACATCTGGCAATTGTTATGGAATACGCATCTGGAGGAGAACTGTTTGAACGTATATGCAATGCTGGCAGGTTTCCAGAAGATGAGGCCCGTTTCTTCTTCCAGCAACTCATTTCAGGAGTCAGCTACTGTCATAATATGCAAGTATGCCATCGTGACTTGAAACTAGAGAACACACTTCTAGATGGAAGCCCAGCTCCTCGTTTAAAGATTTGTGATTTTGGGTACTCCAAGTCATCAGTGCTACATTCACAACCAAAATCCACAGTAGGAACCCCTGCATATATTGCTCCAGAAGTCTTGCTCAAAAAAGAATACGATGGCAAGATAGCAGATGTGTGGTCATGTGGTGTAACATTATACGTCATGTTAGTAGGAGCTTACCCTTTCGAAGACCCCGAAGAACCCAAAAATTTCCGCAAAACCATTCAGCGAATTCTCAATGTCCAATACTCAATTCCATCTTATGTTCATATATCTCCCGAGTGCCGCCATTTAATCTCCCGAATCTTTGTTGCTGATCCCGGCAAGAGAATAAGTATGGATGAAATAAAGAACCATGAATGGTTTAAGAGGAACTTACCGACCGAGTTGACAAAAGAGAACTCGATGGAGGAATTTGGAGGGGCGGATGAGTCGACTCAGAGTGTTGATGAAATAATGCAGATAATTGCGGAAGCTACAATTCCGCCAGCTGGCGCTAATAATTTGAATCAGTATTTGACCGGGAGTTTGGACATTGATGATGACATGGACGAGGATTTGGAGAGTGATCCGGATTTGGATATTGATAGCAGTGGGGAGATAGTTTATGCAATGTAA

TmSnRK2.7(Promoter sequence)

ACAACATACAACAACAACACTTTTTATAAAAAAAAAACATAAATTCCCAAAAAAACTCACATTGCATAAACTATCTCCCCACTGCTATCAATATCCAAATCCGGATCACTTTCCAAATCCTCGTCCATGTCATCATCAATGTCCAAACTCCCGGTCAAATACTGATTCAAATTATTAGCGCCAGCTGGCGGAATTGTAGCTTCCGCAATTATCTGCATTATTTCATCAACACTCTGAGTCGACTCATCCGCCCCTCCAAATTCCTCCATCGAGTTCTCTTTTGTCAACTCGGTTGGTAAGTTCCTCTTAAACCATTCATGGTTCTTTATTTCATCCATACTTATTCTCTGTTTCAATTTTCAAAAATATATAACTTATAAATTACATCCTCAATTTTATAAAATTTTATAAACATATACCTTGGCGGGATCAGCAACAAAGATTCGGGAGATTAAATGGCGGCACTCGGGAGATATATGAACGTAAGATGGAATCGAGTACTGGACATTGAGAATTCTCTGAAAGAAAGTTAAGAAAGGGTAAAATGGTCATTTTACATGGAGGGTATTAATATTAATATAAAAGATATAATTTTAGGTACCTGAATGGTTTTGCGGAAATTTTTGGGTTCTTCGGGGTCTTCGAAAGGGTAAGCTCCTACTAACATGACGTATAATGTTACACCACATGACCACACATCTGCTATCTGGATTTTATCAAGAAAATGTTTTATGTTTTTTTTTTTGAAATGTTTAATTATGAGGGTATAATTGGATTTAATGAAAATTGTTTACCTTGCCATCGTATTCTTTTTTGAGCAAGACTTCTGGAGCAATATATGCAGGGGTTCCTACTGTGGATTTTGGTTGTGAATGTAGCACTGATGACTGATTATTAGAACAAAATAAAGAAAAAAGAAATAAAGATTGGATTTTTATAAGACCCAAGAATCAAGAAAATCAAGAACATCAAGAAAAGTATACCTTGGAGTACCCAAAATCACAAATCTTTAAACGAGGAGCTGGGCTTCCATCTAGAAGTGTGTTCTCTAGTTTCAAGTCACGATGGCATACTTGCTAGATTGTATAAAAGATTAAAAGCATGAATTATAAGTAATTAATAAAATATGTATATATATATATATATATATATATATATATATATATATATATATATATATATATATAAGATGGTAGTTATATACCATATTATGACAGTAGCTGACTCCTGAAATGAGTTGCTGGAAGAAGAAACGTGCCTGGAAGATTTTAAAAAAAAAAAGGAGAAATGGATGCGTTCATATAAGTCAAAAGTCAACACTAGAATTGGAAGTCAATGGATATGGATTTAAGAAGGAACCTCATCTTCTGGAAACCTGCCAGCATTGCATATACGTTCAAACAGTTCTCCTCCAGATGCGTATTCCATAACAATTGCCAGATGTGTTGGTGTTAAGATGACCTGAAGATCAAGTGAAATTAATAATGAAGTCATGAAGAAACAGGTAATTTTTTAACGTAATCAGATACAAACCTCTTTGAATCTGACGATATTTGGATGCCTTAGTGATCTGTGGTTAATGATTTCCCTTTGTACATTTTCGTCAATCTGTTGGACTCGAGATAAGTGAACAATAGTTAGAAGAACTTCAATCACCTCTGTTTATTCGTTACCTTTTACCCCTGTTTTTAGTCTCGAATCACAAGATTGATTGGATTACTCAGTTCGATTTCAAACTATTCTATTACGATCGACGGGAAAAAGCGAAAAAAATGAGAGATAATGTTTAGAAGTGGAAGATGGATACTCGATTATAACACTGACCTTCTCACCTCTCTCGATGTACTTGACTGCAACAAGCTCGTTAGTCTGTTTATCCCTCATCAACCTCGCCACACCAAAATTACCAGAACCAATGTCTCTCACTAGCTCGTAGCGATCACTATCGTGCATGATAGGCATATCCATACCCGGACCCACAGTAAGCGCAGATCGATCCAT

**Table S6. Protein sequence used of AtSnRK2s and OsSAPKs and TmSnRK2 in *T. mongolicum***

TRINITY_DN1952_c0_g1,TmSnRK2.1 MEKYELVKDIGSGNFGVARLMRNKVTKELVAMKYIERGHKIDENVAREIINHRSLRHPNIIRFREVVLTPTHLAIVMEYAAGGELFERIVNAGRFSEDEARYFFQQLISGVHYCHFMQICHRDLKLENTLLDGSPAPRLKICDFGYSKSSLLHSRPKSTVGTPAYIAPEVLSRREYDGKTADVWSCGVTLYVMLVGAYPFEDQEDPKNFRKTINRIMAVQYKIPDYVHISQDCRHLLSRIFVTNASRRITLNEIKTHPWFLKNLPRELTEASQSVYYRKENPTFSPQSVEDIMKIVEEARTPPPVSKSHGAYGWGDDDEDDDDDDGKEAHDGDDDDGDEDEYDKRVKEAHESGEIGPV

TRINITY_DN81_c0_g1,TmSnRK2.2 MVFPPNHVDHPTKAFIVVAYVQTRKLHRALTKTWHQRDFLNRFYDYLLNVMERYEEIKHIGSGNYGVAKLVKDKQSGELYAVKYIERGPKIDEHVEREILNHRSLKHPHIIRFKEVLLTETHLAIVMEYAAGGELFDRIFNAGRFSEDEARFFFQQLISGVSYCHSMQICHRDLKLENVLLDGSSTPRLKICDFGYSKSSVFHSQPKSTVGTPAYIAPEVLSRKEYDGKMADVWSCGVTLYVMLVGAYPFEDEDDPRNFRKTLTRILSVHYSVPDYVRVSVGCKHLLSRIFVANPDKRITIPEIQKHPWFLKELPSELIDEDESISDNDKENNVSQSIKEIKEILQEARRCTLRTKDEGGRMDLEDDIDTDAEIEDDAETSGDFVCAL

TRINITY_DN3720_c0_g1,TmSnRK2.3 MERYELVKEIGSGSFGVAKLVRDKGTRELFAVKFIERGKKIDEHVQREIMNHRSLRHPNIIRFKEVLLTQTHLAIVMEYAAGGELFERICNAGKFSENEARFFFQQLISGVSYCHSMEVCHRDLKLENTLLDGSAAPRVKICDFGYSKSSVLHSQPKSAVGTPAYIAPEVLSRKEYNGKLADVWSCGVTLYVMLVGEYPFGDPDDPRNFRTTVCRILSVQYAIPDTVEISMECKHLLYRIFVANPEKRITIPEIQMHPWFLKNLPTDLMAGGSSNTINAYQSDDEILSIIQEARTHPGMLCDGSPQLLGDSMDFDDLDDSDIEDIDISDDYGCSL

TRINITY_DN15061_c0_g2,TmSnPK2.4 MEICHRDLKLENTLLDGSRSPRLKICDFGYSKSGLLHSQPKSTVGTPAYIAPEVLSRKEYDGKIADVWSCGVTLYVMLVGAYPFEDPRDPRNFRKTIGRIMSVQYSIPDYVRVSKDCRHLLSHIFVANPSKRITIGEIKKHPWFMKNMPKELVEGEKTNYENASRDQSPQGVDELNRIIQEAMNPGEGSTRNGEIVIGDGSLDPEDEIDLDDEIESSGDYGGQG

TRINITY_DN27171_c0_g1,TmSnPK2.5 MEEKYEPVKDGFGNFEVARLVRDKKTKEVFAVIYIDRGEKIDEKVHKEIINHKLLRHPNIVRFKEVLLTQKRLAIVMEYAHGGELFRNIRSRGRFSEDEARVLFQQLISGVNYCHSMGICHRDLKLENNLLDGSPSPRLKIFDFGHSKFGLLEPQAKSRGTPAYIAPEVLSRKEYDGKITDVWACGVTLYVMLVGAYPFEDPEDSRNFRKSMERIMNVQYSIPDDVCVSVDCRHLLSHILLANPDKRITIAEIKKHPWFMKNMPKDLVEGEKTNYENASFDQSLQSVDEVNHIIQMAKVPGEGSTTRDGREEIGGSMDPDDDDDDDDFDLQNEIDYSAQM

TRINITY_DN2250_c0_g1_i2,TmSnRK2.6 MDRSALTMGPGMDMPIMHDSDRYELVKDIGSGNFGVARLMRDKLSNELVAVKYIERGEKIDENVQREIINHRSLRHPNIVRFKEVILTPTHLAIVMEYASGGELFERICNAGRFPEDEARFFFQQLVSGVGYCHNMQVCHRDLKLENTLLDGSQAPRLKICDFGYSKSSVLHSQPKSTVGTPAYIAPEVLLKKEYDGKIADVWSCGVTLYVMLVGAYPFEDPEEPKNFRNTIQRILNVQYSIPAYVHISTPCRNLISRIFVAEPAKRITMDEIRNHEWFIRNLPGDLMNENAMDQFRGPDQPTQSVDEIMQIIAEATIPPAGAHNLNQYLTGSLDIDDDMDEDLESDPDLDIDSSGEIVYAL

TRINITY_DN2250_c0_g1_i5,TmSnRK2.7 MDRSALTVGPGMDMPIMHDSDRYELVRDIGSGNFGVARLMRDKQTNELVAVKYIERGEKIDENVQREIINHRSLRHPNIVRFKEVILTPTHLAIVMEYASGGELFERICNAGRFPEDEARFFFQQLISGVSYCHNMQVCHRDLKLENTLLDGSPAPRLKICDFGYSKSSVLHSQPKSTVGTPAYIAPEVLLKKEYDGKIADVWSCGVTLYVMLVGAYPFEDPEEPKNFRKTIQRILNVQYSIPSYVHISPECRHLISRIFVADPAKRISMDEIKNHEWFKRNLPTELTKENSMEEFGGADESTQSVDEIMQIIAEATIPPAGANNLNQYLTGSLDIDDDMDEDLESDPDLDIDSSGEIVYAM

XP_023758923.1,LsSAPK3 MEEKYEPLKELGSGNFGVARLVRDKKTKELFAVKYIERGKKIDENVQREIINHRSLRHPNIVRFKEVLLTRTHLAIVMEYAAGGELFSKITSAGRFSEDEARFFFQQLLSGVSYCHSMEICHRDLKLENTLLDGSPSPRLKICDFGYSKSGLLHSQPKSTVGTPAYIAPEVLSRKEYDGKIADVWSCGVTLYVMLVGAYPFEDPEDPRNFRKTIGRIVSVQYSIPDYVRVSVDCRHLLSHIFVANPSKRITIPEIKKHPWFVKNMPKDLVEGEKTNYENASFDQSLQSVEEVNRIIQEAKVPGEGSTTTDGRPEIGGSMDPDEDDFDLENEIDYSGDHSAQI

P43292,AtSnRK2.1 MDKYDVVKDLGAGNFGVARLLRHKDTKELVAMKYIERGRKIDENVAREIINHRSLKHPNIIRFKEVILTPTHLAIVMEYASGGELFDRICTAGRFSEAEARYFFQQLICGVDYCHSLQICHRDLKLENTLLDGSPAPLLKICDFGYSKSSILHSRPKSTVGTPAYIAPEVLSRREYDGKHADVWSCGVTLYVMLVGAYPFEDPNDPKNFRKTIQRIMAVQYKIPDYVHISQECKHLLSRIFVTNSAKRITLKEIKNHPWYLKNLPKELLESAQAAYYKRDTSFSLQSVEDIMKIVGEARNPAPSTSAVKSSGSGADEEEEEDVEAEVEEEEDDEDEYEKHVKEAQSCQESDKA

Q39192.1,AtSnRK2.2 MDPATNSPIMPIDLPIMHDSDRYDFVKDIGSGNFGVARLMTDRVTKELVAVKYIERGEKIDENVQREIINHRSLRHPNIVRFKEVILTPSHLAIVMEYAAGGELYERICNAGRFSEDEARFFFQQLISGVSYCHAMQICHRDLKLENTLLDGSPAPRLKICDFGYSKSSVLHSQPKSTVGTPAYIAPEILLRQEYDGKLADVWSCGVTLYVMLVGAYPFEDPQEPRDYRKTIQRILSVTYSIPEDLHLSPECRHLISRIFVADPATRITIPEITSDKWFLKNLPGDLMDENRMGSQFQEPEQPMQSLDTIMQIISEATIPTVRNRCLDDFMADNLDLDDDMDDFDSESEIDVDSSGEIVYAL

Q39193.1,AtSnRK2.3 MDRAPVTTGPLDMPIMHDSDRYDFVKDIGSGNFGVARLMRDKLTKELVAVKYIERGDKIDENVQREIINHRSLRHPNIVRFKEVILTPTHLAIIMEYASGGELYERICNAGRFSEDEARFFFQQLLSGVSYCHSMQICHRDLKLENTLLDGSPAPRLKICDFGYSKSSVLHSQPKSTVGTPAYIAPEVLLRQEYDGKIADVWSCGVTLYVMLVGAYPFEDPEEPRDYRKTIQRILSVKYSIPDDIRISPECCHLISRIFVADPATRISIPEIKTHSWFLKNLPADLMNESNTGSQFQEPEQPMQSLDTIMQIISEATIPAVRNRCLDDFMTDNLDLDDDMDDFDSESEIDIDSSGEIVYAL

P43291.1,AtSnRK2.4 MDKYELVKDIGAGNFGVARLMKVKNSKELVAMKYIERGPKIDENVAREIINHRSLRHPNIIRFKEVVLTPTHLAIAMEYAAGGELFERICSAGRFSEDEARYFFQQLISGVSYCHAMQICHRDLKLENTLLDGSPAPRLKICDFGYSKSSLLHSRPKSTVGTPAYIAPEVLSRREYDGKMADVWSCGVTLYVMLVGAYPFEDQEDPKNFRKTIQKIMAVQYKIPDYVHISQDCKNLLSRIFVANSLKRITIAEIKKHSWFLKNLPRELTETAQAAYFKKENPTFSLQTVEEIMKIVADAKTPPPVSRSIGGFGWGGNGDADGKEEDAEDVEEEEEEVEEEEDDEDEYDKTVKEVHASGEVRIS

Q9FFP9.1 ,AtSnRK2.5 MDKYEVVKDLGAGNFGVARLLRHKETKELVAMKYIERGRKIDENVAREIINHRSLRHPNIIRFKEVILTPTHLAIVMEYASGGELFERICNAGRFSEAEARYFFQQLICGVDYCHSLQICHRDLKLENTLLDGSPAPLLKICDFGYSKSSLLHSRPKSTVGTPAYIAPEVLSRREYDGKHADVWSCGVTLYVMLVGGYPFEDPDDPRNFRKTIQRIMAVQYKIPDYVHISQECRHLLSRIFVTNSAKRITLKEIKKHPWYLKNLPKELTEPAQAAYYKRETPSFSLQSVEDIMKIVGEARNPAPSSNAVKGFDDDEEDVEDEVEEEEEEEEEEEEEEEEEEDEYEKHVKEAHSCQEPPKA

Q940H6.1,AtSnRK2.6 MDRPAVSGPMDLPIMHDSDRYELVKDIGSGNFGVARLMRDKQSNELVAVKYIERGEKIDENVKREIINHRSLRHPNIVRFKEVILTPTHLAIVMEYASGGELFERICNAGRFSEDEARFFFQQLISGVSYCHAMQVCHRDLKLENTLLDGSPAPRLKICDFGYSKSSVLHSQPKSTVGTPAYIAPEVLLKKEYDGKVADVWSCGVTLYVMLVGAYPFEDPEEPKNFRKTIHRILNVQYAIPDYVHISPECRHLISRIFVADPAKRISIPEIRNHEWFLKNLPADLMNDNTMTTQFDESDQPGQSIEEIMQIIAEATVPPAGTQNLNHYLTGSLDIDDDMEEDLESDLDDLDIDSSGEIVYAM

Q9SMQ4.1,AtSnRK2.7 MERYDILRDLGSGNFGVAKLVREKANGEFYAVKYIERGLKIDEHVQREIINHRDLKHPNIIRFKEVFVTPTHLAIVMEYAAGGELFERICNAGRFSEDEGRYYFKQLISGVSYCHAMQICHRDLKLENTLLDGSPSSHLKICDFGYSKSSVLHSQPKSTVGTPAYVAPEVLSRKEYNGKIADVWSCGVTLYVMLVGAYPFEDPEDPRNIRNTIQRILSVHYTIPDYVRISSECKHLLSRIFVADPDKRITVPEIEKHPWFLKGPLVVPPEEEKCDNGVEEEEEEEEKCRQSVEEIVKIIEEARKGVNGTDNNGGLGLIDGSIDLDDIDDADIYDDVDDDEERNGDFVCAL

Q9M9E9.1,AtSnRK2.8 MERYEIVKDIGSGNFGVAKLVRDKFSKELFAVKFIERGQKIDEHVQREIMNHRSLIHPNIIRFKEVLLTATHLALVMEYAAGGELFGRICSAGRFSEDEARFFFQQLISGVNYCHSLQICHRDLKLENTLLDGSEAPRVKICDFGYSKSGVLHSQPKTTVGTPAYIAPEVLSTKEYDGKIADVWSCGVTLYVMLVGAYPFEDPSDPKDFRKTIGRILKAQYAIPDYVRVSDECRHLLSRIFVANPEKRITIEEIKNHSWFLKNLPVEMYEGSLMMNGPSTQTVEEIVWIIEEARKPITVATGLAGAGGSGGSSNGAIGSSSMDLDDLDTDFDDIDTADLLSPL

O64812.1,AtSnRK2.9 MEKYEMVKDLGFGNFGLARLMRNKQTNELVAVKFIDRGYKIDENVAREIINHRALNHPNIVRFKEVVLTPTHLGIVMEYAAGGELFERISSVGRFSEAEARYFFQQLICGVHYLHALQICHRDLKLENTLLDGSPAPRLKICDFGYSKSSVLHSNPKSTVGTPAYIAPEVFCRSEYDGKSVDVWSCGVALYVMLVGAYPFEDPKDPRNFRKTVQKIMAVNYKIPGYVHISEDCRKLLSRIFVANPLHRSTLKEIKSHAWFLKNLPRELKEPAQAIYYQRNVNLINFSPQRVEEIMKIVGEARTIPNLSRPVESLGSDKKDDDEEEYLDANDEEWYDDYA

Q9C958.1 ,AtSnRK2.10 MDKYELVKDIGAGNFGVARLMRVKNSKELVAMKYIERGPKIDENVAREIINHRSLRHPNIIRFKEVVLTPTHIAIAMEYAAGGELFERICSAGRFSEDEARYFFQQLISGVSYCHAMQICHRDLKLENTLLDGSPAPRLKICDFGYSKSSLLHSMPKSTVGTPAYIAPEVLSRGEYDGKMADVWSCGVTLYVMLVGAYPFEDQEDPKNFKKTIQRIMAVKYKIPDYVHISQDCKHLLSRIFVTNSNKRITIGDIKKHPWFLKNLPRELTEIAQAAYFRKENPTFSLQSVEEIMKIVEEAKTPARVSRSIGAFGWGGGEDAEGKEEDAEEEVEEVEEEEDEEDEYDKTVKQVHASMGEVRVS

Q75LR7.1,OsSAPK1 MERYEVMRDIGSGNFGVAKLVRDVATNHLFAVKFIERGLKIDEHVQREIMNHRSLKHPNIIRFKEVVLTPTHLAIVMEYAAGGELFERICNAGRFSEDEARFFFQQLISGVSYCHSMQVCHRDLKLENTLLDGSVTPRLKICDFGYSKSSVLHSQPKSTVGTPAYIAPEVLSRKEYDGKVADVWSCGVTLYVMLVGAYPFEDPDDPRNFRKTITRILSVQYSIPDYVRVSADCRHLLSRIFVGNPEQRITIPEIKNHPWFLKNLPIEMTDEYQRSMQLADMNTPSQSLEEVMAIIQEARKPGDAMKLAGAGQVACLGSMDLDDIDDIDDIDIENSGDFVCAL

Q0D4J7.1 ,OsSAPK2 MERYEVIKDIGSGNFGVAKLVRDVRTKELFAVKFIERGQKIDENVQREIMNHRSLRHPNIVRFKEVVLTPTHLAIVMEYAAGGELFERICSAGRFSEDEARFFFQQLISGVSYCHSMQICHRDLKLENTLLDGSIAPRLKICDFGYSKSSLLHSQPKSTVGTPAYIAPEVLARKEYDGKVADVWSCGVTLYVMLVGAYPFEDPDEPRNFRKTITRILSVQYMVPDYVRVSMECRHLLSRIFVANPEQRITIPEIKNHPWFLKNLPIEMTDEYQMSVQMNDINTPSQGLEEIMAIIQEARKPGDGSKFSGQIPGLGSMELDDVDTDDIDVEDSGDFVCAL

P0C5D6.1 ,OsSAPK3 MEERYEALKELGAGNFGVARLVRDKRSKELVAVKYIERGKKIDENVQREIINHRSLRHPNIIRFKEVCLTPTHLAIVMEYAAGGELFEQICTAGRFSEDEARYFFQQLISGVSYCHSLEICHRDLKLENTLLDGSPTPRVKICDFGYSKSALLHSKPKSTVGTPAYIAPEVLSRKEYDGKVADVWSCGVTLYVMLVGSYPFEDPGDPRNFRKTISRILGVQYSIPDYVRVSSDCRRLLSQIFVADPSKRITIPEIKKHTWFLKNLPKEISEREKADYKDTDAAPPTQAVEEIMRIIQEAKVPGDMAAADPALLAELAELKSDDEEEAADEYDTY

Q5N942.2,OsSAPK4 MEKYEAVRDIGSGNFGVARLMRNRETRELVAVKCIERGHRIDENVYREIINHRSLRHPNIIRFKEVILTPTHLMIVMEFAAGGELFDRICDRGRFSEDEARYFFQQLICGVSYCHHMQICHRDLKLENVLLDGSPAPRLKICDFGYSKSSVLHSRPKSAVGTPAYIAPEVLSRREYDGKLADVWSCGVTLYVMLVGAYPFEDQDDPKNIRKTIQRIMSVQYKIPDYVHISAECKQLIARIFVNNPLRRITMKEIKSHPWFLKNLPRELTETAQAMYYRRDNSVPSFSDQTSEEIMKIVQEARTMPKSSRTGYWSDAGSDEEEKEEEERPEENEEEEEDEYDKRVKEVHASGELRMSSLRI

Q7XKA8.1,OsSAPK5 MEKYEPVREIGAGNFGVAKLMRNKETRELVAMKFIERGNRIDENVFREIVNHRSLRHPNIIRFKEVVVTGRHLAIVMEYAAGGELFERICEAGRFHEDEARYFFQQLVCGVSYCHAMQICHRDLKLENTLLDGSPAPRLKICDFGYSKSSLLHSRPKSTVGTPAYIAPEVLSRREYDGKLADVWSCGVTLYVMLVGAYPFEDPKDPKNFRKTISRIMSVQYKIPEYVHVSQPCRHLLSRIFVANPYKRISMGEIKSHPWFLKNLPRELKEEAQAVYYNRRGADHAASSASSAAAAAAFSPQSVEDIMRIVQEAQTVPKPDKPVSGYGWGTDDDDDDQQPAEEEDEEDDYDRTVREVHASVDLDMSNLQIS

Q6ZI44.1,OsSAPK6 MEKYELLKDIGSGNFGVARLMRNRETKELVAMKYIPRGLKIDENVAREIINHRSLRHPNIIRFKEVVLTPTHLAIVMEYAAGGELFDRICSAGRFSEDESRYFFQQLICGVSYCHFMQICHRDLKLENTLLDGSPAPRLKICDFGYSKSSLLHSKPKSTVGTPAYIAPEVLSRREYDGKMADVWSCGVTLYVMLVGAYPFEDPDDPKNFRKTIGRIVSIQYKIPEYVHISQDCRQLLSRIFVANPAKRITIREIRNHPWFMKNLPRELTEAAQAKYYKKDNSARTFSDQTVDEIMKIVQEAKTPPPSSTPVAGFGWTEEEEQEDGKNPDDDEGDRDEEEGEEGDSEDEYTKQVKQAHASCDLQKS

Q7XQP4.2,OsSAPK7 MERYELLKDIGAGNFGVARLMRNKETKELVAMKYIPRGLKIDENVAREIINHRSLRHPNIIRFKEVVVTPTHLAIVMEYAAGGELFDRICNAGRFSEDEARYFFQQLICGVSYCHFMQICHRDLKLENTLLDGSPAPRLKICDFGYSKSSLLHSKPKSTVGTPAYIAPEVLSRREYDGKTADVWSCGVTLYVMLVGAYPFEDPDDPKNFRKTIGRIMSIQYKIPEYVHVSQDCRQLLSRIFVANPAKRITIREIRNHPWFLKNLPRELTEAAQAMYYKKDNSAPTYSVQSVEEIMKIVEEARTPPRSSTPVAGFGWQEEDEQEDNSKKPEEEQEEEEDAEDEYDKQVKQVHASGEFQLS

Q7Y0B9.1,OsSAPK8 MAAAGAGAGAPDRAALTVGPGMDMPIMHDSDRYELVRDIGSGNFGVARLMRDRRTMELVAVKYIERGEKIDDNVQREIINHRSLKHPNIIRFKEVILTPTHLAIVMEYASGGELFERICKNVRFSEDEARYFFQQLISGVSYCHSMQVCHRDLKLENTLLDGSPAPRLKICDFGYSKSSVLHSQPKSTVGTPAYIAPEVLLKKEYDGKTADVWSCGVTLYVMVVGAYPFEDPEEPKNFRKTIQRILNVQYSIPENVDISPECRHLISRIFVGDPSLRITIPEIRSHGWFLKNLPADLMDDDSMSSQYEEPDQPMQTMDQIMQILTEATIPPACSRINHILTDGLDLDDDMDDLDSDSDIDVDSSGEIVYAM

Q75V57.1,OsSAPK9 MERAAAGPLGMEMPIMHDGDRYELVKEIGSGNFGVARLMRNRASGDLVAVKYIDRGEKIDENVQREIINHRSLRHPNIIRFKEVILTPTHLAIVMEYASGGELFERICSAGRFSEDEARFFFQQLISGVSYCHSMQVCHRDLKLENTLLDGSTAPRLKICDFGYSKSSVLHSQPKSTVGTPAYIAPEVLLKKEYDGKIADVWSCGVTLYVMLVGAYPFEDPEDPKNFRKTIQKILGVQYSIPDYVHISPECRDLITRIFVGNPASRITMPEIKNHPWFMKNIPADLMDDGMVSNQYEEPDQPMQNMNEIMQILAEATIPAAGTSGINQFLTDSLDLDDDMEDMDSDLDLDIESSGEIVYAM

Q75H77.1,OsSAPK10 MDRAALTVGPGMDMPIMHDGDRYELVRDIGSGNFGVARLMRSRADGQLVAVKYIERGDKIDENVQREIINHRSLRHPNIIRFKEVILTPTHLAIVMEYASGGELFERICNAGRFSEDEARFFFQQLISGVSYCHSMQVCHRDLKLENTLLDGSTAPRLKICDFGYSKSSVLHSQPKSTVGTPAYIAPEVLLKKEYDGKIADVWSCGVTLYVMLVGAYPFEDPDEPKNFRKTIQRILGVQYSIPDYVHISPECRDLIARIFVANPATRISIPEIRNHPWFLKNLPADLMDDSKMSSQYEEPEQPMQSMDEIMQILAEATIPAAGSGGINQFLNDGLDLDDDMEDLDSDPDLDVESSGEIVYAM

**Table S7. Raw data of tissue and ABA treatment qRT-PCR results**

| **samples** | **GADPH** | **β-Actin** | **2.1** | **2.2** | **2.3** | **2.4** | **2.5** | **2.6** | **2.7** |
| --- | --- | --- | --- | --- | --- | --- | --- | --- | --- |
| root | 17.1 | 16.07 | 20.63 | 20.77 | 21.06 | 31.54 | 26.55 | 21.1 | 21.51 |
| root | 16.67 | 16 | 20.24 | 20.43 | 21.07 | 31.82 | 27.26 | 20.49 | 21.19 |
| root | 16.54 | 15.74 | 20.27 | 20.76 | 21.01 | 32.76 | 27.31 | 20.16 | 21.5 |
| leaf | 17.43 | 17.22 | 21.02 | 21.17 | 21.4 | 27.65 | 34.53 | 20.75 | 20.64 |
| leaf | 18.52 | 17.18 | 21.21 | 21.29 | 21.43 | 27.32 | 34.67 | 20.65 | 20.54 |
| leaf | 17.09 | 17.21 | 21.18 | 21.39 | 21.3 | 27.56 | 34.81 | 20.26 | 20.94 |
| flower | 15.83 | 16.49 | 19.87 | 21 | 21.16 | 29.04 | 30.5 | 19.65 | 21.08 |
| flower | 15.97 | 16.51 | 19.51 | 21.17 | 21.44 | 28.99 | 32.17 | 20.1 | 21 |
| flower | 15.6 | 16.41 | 19.71 | 21.13 | 21.38 | 29.05 | 29.69 | 19.5 | 21.14 |
| 0 h | 15.3 | 16.42 | 21.05 | 21.43 | 21.67 | 23.13 | 25.39 | 19.69 | 28.68 |
| 0 h | 15.32 | 16.6 | 20.52 | 21.6 | 21.31 | 22.57 | 25.61 | 20.16 | 29.06 |
| 0 h | 15.38 | 16.03 | 21.1 | 21.89 | 22.05 | 23.2 | 25.74 | 20.35 | 28.94 |
| 2 h | 15.23 | 15.93 | 20.37 | 21.3 | 21.67 | 19.82 | 25.75 | 19.3 | 29.36 |
| 2 h | 15.68 | 16.33 | 20.2 | 21.13 | 21.66 | 20.08 | 25.53 | 19.32 | 29.24 |
| 2 h | 15.24 | 16.04 | 20.57 | 21.2 | 21.35 | 19.82 | 25.74 | 20.14 | 29.27 |
| 4 h | 15.89 | 16.23 | 20.64 | 21.09 | 21.48 | 19.15 | 24.66 | 19.81 | 29.09 |
| 4 h | 15.46 | 16.11 | 20.83 | 20.81 | 21.64 | 19.1 | 24.92 | 19.41 | 29.1 |
| 4 h | 15.59 | 15.94 | 20.67 | 21.1 | 22.1 | 19.42 | 24.47 | 20.22 | 28.89 |
| 8 h | 15.57 | 16.19 | 21.23 | 21.3 | 21.08 | 20 | 24.45 | 19.57 | 28.62 |
| 8 h | 15.77 | 16.6 | 20.38 | 21.13 | 21.27 | 19.76 | 24.84 | 19.6 | 29.04 |
| 8 h | 15.44 | 16.46 | 20.85 | 21.05 | 21.13 | 19.96 | 24.66 | 19.33 | 28.66 |
| 24 h | 15.68 | 16.2 | 20.68 | 21.33 | 21.9 | 20.56 | 26.5 | 19.45 | 29.9 |
| 24 h | 15.48 | 15.56 | 20.5 | 21.3 | 22.06 | 20.51 | 26.44 | 19.24 | 30.09 |
| 24 h | 15.65 | 16.16 | 20.79 | 21.53 | 21.89 | 20.89 | 26.22 | 19.97 | 30.43 |
